# Structural connectome changes as biomarkers of stroke recovery: a longitudinal 7T MRI study

**DOI:** 10.1186/s12868-026-01010-9

**Published:** 2026-05-14

**Authors:** Esin Avci-Colak, Gitta Rohweder, Riccardo Iandolo, Giulia Bommarito, Ioanna Sandvig, Axel Sandvig

**Affiliations:** 1 Department of Neuromedicine and Movement Science, Faculty of Medicine and Health Sciences, Norwegian University of Science and Technology (NTNU), Trondheim, Norway; 2 Stroke Unit, Department of Medicine, St. Olav’s University Hospital, Trondheim, Norway; 3 Department of Diagnostic Imaging, IRCCS SYNLAB, Naples, Italy; 4 Department of Clinical Neurosciences, Lausanne University Hospital and University of Lausanne, Lausanne, Switzerland; 5 Department of Community Medicine and Rehabilitation, Umeå University, Umeå, Sweden; 6 National Highly Specialized Care for Spinal Cord Injuries, Umeå University Hospital, Umeå, Sweden

**Keywords:** Structural connectivity, High-field MRI, Ischemia, Neural network, Plasticity

## Abstract

**Background:**

Stroke leads to widespread brain connectivity changes, impacting areas both close and remote to the lesion. Post-stroke recovery dynamics are not fully understood. Investigating structural network reorganization over time can thus provide valuable information on adaptive and maladaptive neural plasticity changes on a subject-specific level.

**Methods:**

Four first-time ischemic stroke patients (3 M, aged 50–69 years) with upper-extremity motor impairment were examined using ultra-high field 7T MRI structural imaging protocols. For each patient, we performed longitudinal lesion quantification and white matter connectivity analysis at three critical timepoints associated with post-stroke recovery: within 1 week, at ~ 1 month, and at ~ 3 months. Using the structural MRI images, we generated patient-specific weighted structural connectivity matrices at each timepoint. We utilized the Schaefer-Yeo and Automated Anatomical Labeling atlases to derive both anatomical regions and resting-state networks based on pre-defined parcel assignments. We examined lesion evolution and white matter connectivity changes as disconnections, re-emerging connections, and existing connections with an increase in estimated connectivity strength over time. We further conducted exploratory edge-level analyses to examine the connectivity strength changes for each patient.

**Results:**

Across all patients and timepoints, an increase in estimated connectivity strength of pre-existing connections dominated structural reorganization. Temporally, the four patients revealed distinct neural reorganization patterns. Patient 1 exhibited robust structural changes in the late ~1 to ~ 3 months stage, whereas Patient 2 in the early < 1 week to ~ 1 month stage. Patient 3 had continuous network growth, while Patient 4 demonstrated stable network reorganization. In our sample, the somatomotor and attention networks underwent the most dynamic reorganization. Somatomotor and salience/ventral attention regions exhibited increased connectivity strength, and in cortical stroke cases, dorsal attention regions demonstrated decreased connectivity strength.

**Conclusions:**

In this longitudinal case series, post-stroke neural network reorganization appears to be driven by an increase in estimated connectivity strength of surviving white matter connections, suggesting compensatory neuroplasticity. Adaptive changes were most evident in the somatomotor and salience/ventral attention networks within this sample, while the dorsal attention network suggested a more limited contribution to adaptive network changes. Individual differences in the timing and pattern or reorganization highlight the potential need for further research into personalized treatment approaches to promote adaptive recovery.

**Supplementary information:**

The online version contains supplementary material available at 10.1186/s12868-026-01010-9.

## Introduction

Stroke is a major global health issue, contributing to long-term disability and mortality worldwide, with ischemic stroke accounting for ca. 90% of cases [1]. Despite advances in medicine, stroke treatment remains highly time-sensitive and only a small group of patients are eligible for acute treatments such as recanalization in the case of an occluding thrombus [2, 3]. Recovery patterns differ substantially among stroke survivors, with some facing lasting neurological challenges while others achieve partial or full motor recovery [4, 5]. This variability in outcomes may reflect underlying patient differences. Even clinically similar strokes can vary in lesion location and load, baseline severity, coexisting medical conditions, and genetic factors [4, 6–8]. The exact mechanisms underlying this variability are poorly understood. Timing is suggested to play a crucial role, as spontaneous recovery typically reaches its maximum and functional improvements plateau after three months for the majority of patients [4, 9, 10]. Within the first week, the changes in inhibitory neurotransmission peak and early clinical assessments can predict functional recovery [11–13], whereas around one month, maximum changes in motor function occur [10, 14]. A critical window with regards to neural plasticity is suggested to last around three months, with motor rehabilitation being most effective within that time frame [15]. Studying patient-level recovery trajectories at key timepoints within this three-month window could yield important insights into the adaptive neural reorganization and support personalized treatment and rehabilitation strategies.

Ischemic stroke lesions initially cause focal structural damage through neuronal cell and tissue death in the affected vascular territory [16]. However, the impact often extends far beyond the lesion site, as lesions commonly damage white matter structures along with the grey matter [17]. This widespread structural damage affects distant brain regions and disrupts neural communication through large-scale brain networks [18, 19]. Neural communication does not only rely on preserved connections, but also on the integrity of multi-synaptic pathways [20]. Stroke lesions can result in direct loss of white matter connections between brain areas, or indirectly disrupt the intermediate connections required for inter-regional communication. Both forms of damage are shown to affect post-stroke behavioral outcomes [21, 22]. For instance, post-stroke motor function impairment is highly common among stroke survivors [23, 24], even when lesions do not involve core motor regions. Motor activities are primarily facilitated by the primary motor cortex, primary somatosensory cortex and supplementary motor areas [25–28], where size and severity of stroke lesions affect contralesional motor cortex reorganization [29]. Subcortical lesions, e.g. those affecting basal ganglia or internal capsule, can lead to motor impairment due to disruptions in critical white matter tracts or multi-synaptic loops like basal ganglia-thalamo-cortical circuits [30, 31]. Therefore, understanding how structural damage propagates across the entire network would provide valuable insights into the relationship between lesion characteristics and different functional outcomes.

Stroke as a network disorder [17], triggers widespread structural and functional neural network reorganization to compensate for the lesion-induced damage [32]. Neuroplasticity mechanisms start minutes after stroke onset [33], and the entire neural network undergoes complex changes that can either promote functional recovery, or contribute to persistent neurological impairment [4, 17, 34]. The location and extent of the infarct, or lesion load, are among the key factors influencing the need for neural compensation [35] and are closely associated with clinical outcomes [36]. Neural plasticity mechanisms could be triggered spontaneously or through therapy and involve dendrite and axon growth to establish new local and global connections [4, 37]. However, plasticity may not always be adaptive, and mechanisms such as competitive interaction between brain hemispheres can have a negative impact on recovery [38]. Therefore, investigating structural reorganization post-stroke is essential for understanding how network-level changes mediate recovery and for identifying potential targets for clinical interventions to promote adaptive plasticity.

Advanced neuroimaging approaches offer promising tools to examine post-stroke network changes. One powerful method is the connectome analysis, which characterizes structural links between brain areas using magnetic resonance imaging (MRI) [39, 40]. Structural connectivity encompasses the anatomical connections among brain regions and is believed to fundamentally influence functional connectivity, although the precise structure–function relationship remains incompletely understood. [41]. The integration of lesion analysis with a connectome approach has been suggested to yield more extensive insights into brain function and behavioral symptoms [42]. Furthermore, ultra-high field MRI imaging offers valuable advantages over conventional MRI field strengths, as greater spatial resolution and tissue contrast enable more sensitivity to microstructural white matter changes and detailed mapping of lesions [43, 44]. Integrating ultra-high field imaging with parcel-level connectome analysis thus provides a unique perspective to study widespread lesion effects across the neural network and distributed large-scale network regions with increased detail and precision. Within this framework, investigating patient-specific trajectories is critical, as group-level analyses obscure individual variabilities by averaging effects across patients, thereby limiting precision in diagnosis and intervention [45]. This creates a gap in understanding the mechanisms that drive different recovery trajectories following stroke. As long-term recovery depends not only on the initial lesion but also on how surviving white matter pathways adapt following injury [46], characterizing patient-specific connectome reorganization during the early recovery period could help identify adaptive and maladaptive network changes. This could ultimately support the development of timely and personalized rehabilitation strategies.

In this longitudinal case series, we examined four first-time ischemic stroke patients with upper-extremity motor impairments over time, both clinically and with MRI at the following three timepoints: (i) within 1 week, (ii) at ~ 1 month, and (iii) at ~ 3 months. By integrating ultra-high field 7T MRI with parcel-level connectome analysis, we aimed to characterize early structural network reorganization longitudinally after stroke. We analyzed white matter connectivity changes at both the global structural network level and within anatomically defined resting-state network (RSN) clusters. This approach aimed to reveal how neural pathways may adapt through compensatory rewiring and strengthened connections or fail to achieve recovery due to unresolved disconnections and decreased connectivity strength. We aimed to answer the following research questions: 1) Do the structural connectomes of patients exhibit distinct temporal patterns of reorganization? 2) Are there common mechanisms driving post-stroke structural reorganization? 3) How do different distributed network regions contribute to structural network recovery? As our sample included patients with motor symptoms, we anticipated that the somatomotor network regions would exhibit the most pronounced adaptive connectivity changes.

## Materials and methods

### Participants

Four first-time ischemic stroke patients (3 M, aged 50–69 years) with upper-extremity motor impairment were included. Inclusion criteria followed as: 1) first-time cortical or subcortical ischemic stroke, 2) hospital admission within 24 hours of stroke onset, 3) upper-extremity paresis and/or reduced hand function, 4) ability to provide informed consent, 5) ability to be scanned at 7T (no metallic implants, neurostimulator, pacemaker, claustrophobia). Exclusion criteria were: 1) history of transient ischemic attack, 2) aphasia, 3) pre-stroke dependence on daily activities (modified Rankin scale ≥ 3), 4) previous stroke, neurotrauma, or brain tumor, 5) cognitive dysfunction, 6) prior neurological, neurodegenerative, psychiatric, or musculoskeletal conditions. Eligibility was confirmed by a senior medical doctor (G.R.) working at the Stroke Unit, St. Olav’s Hospital, Trondheim. Data were collected at three timepoints from post-stroke onset: within 7 days (S1), at ~ 1 month (S2), and at ~ 3 months (S3). Exact assessment times are noted in Table 1. Clinical assessments were performed at each time point, including: modified Rankin scale (mRS) for global disability [47, 48], National Institutes of Health Stroke Scale (NIHSS) for stroke severity [49, 50], Barthel Index (BI) for activities of daily living [51], and Fugl-Meyer Assessment – Upper Extremity (FMA-UE) for motor function [52, 53], including the components of Upper Extremity, Wrist, Hand, and Coordination/Speed.

**Table 1:** Demographic, lesion location, timing of MRI/clinical assessments, and scores of the patients. For modified Rankin scale and National Institutes of Health stroke scale, lower scores indicate better functional recovery. For Barthel index and Fugl-Meyer assessment – upper extremity, higher scores indicate better functional recovery

		Patient 1	Patient 2	Patient 3	Patient 4
Demographics					
Age (years)		60	66	69	50
Sex (male/female)		M	F	M	M
Stroke location		Left temporal lobe	Right fronto-parietal lobe	Right caudate nucleus	Left caudate nucleus
Time of assessments (days after stroke onset)					
First assessment (S1)		5 days	7 days	3 days	2 days
Second assessment (S2)		40 days	42 days	33 days	35 days
Third assessment (S3)		82 days	105 days	92 days	107 days
Clinical assessments					
modified Rankin scale (0–5)	S1	2	3	4	2
	S2	2	2	4	2
	S3	2	2	2	1
National institutes of health stroke scale (0–42)	S1	3	6	5	1
	S2	2	1	6	1
	S3	2	2	3	0
Barthel Index (0–100)	S1	85	60	50	100
	S2	100	95	65	100
	S3	100	95	100	100
Motor assessment					
Fugl-Meyer assessment – Upper Extremity (0–66)	S1	62	35	58	66
	S2	66	57	59	66
	S3	66	56	66	65

### MRI data collection, processing and lesion segmentation

Neuroimaging data were acquired using a 7T MRI scanner. High-resolution T1-weighted images were obtained using 3D-MP2RAGE sequence and T2-weighted images using 3D-FLAIR sequence, both with 0.75 mm isotropic resolution. Structural preprocessing of T1-weighted and T2-FLAIR MRI images followed the procedures detailed in our previous work [54]. Details on the acquisition parameters and preprocessing steps can be found in Supplementary Materials.

An expert neurologist (G.B) manually segmented stroke lesions on the preprocessed images, generating binary lesion masks at 0.75 mm isotropic native resolution. To ensure accurate registration while preserving lesion volume and boundaries, we applied a two-step registration procedure. First, native-space lesion masks were affine-registered to a high-resolution 0.7 mm isotropic MNI template using FSL tools [55]. We then implemented an enantiomorphic normalization approach to prevent distortion of lesioned tissue during non-linear registration. By isolating the healthy tissue from the contralesional hemisphere and mirroring it to fill the lesioned area in the ipsilesional hemisphere, an enantiomorphic-filled T1 image was created. The second step of the registration involved Advanced Normalization Tools (ANTs) symmetric diffeomorphic registration to align the images and lesion masks to 1 mm isotropic Montreal Neurological Institute (MNI) space, which is required for the Lesion Quantification Toolkit (LQT) utilized in the later stages [22]. The registration quality was checked using normalized mutual information (NMI) between the patient-specific warped images and the MNI template at each session. All values were stable across sessions with a maximum within-patient deviation of ≤0.0069 (Supplementary Table 1, left panel). This two-step approach was done to ensure preserving the lesion border and size accuracy, and lesion alignment was verified at all stages. More details are provided in Supplementary Materials.

### Data analysis

#### Structural network construction

We used the Schaefer 2018 cortical parcellation with 1000 parcels and 17 resting-state networks (RSNs) [56], combined with Automated Anatomical Labeling (AAL) atlas [57] with additional 35 subcortical parcels and 4 RSNs. Natively integrated within the LQT, a modified parcellation combines these two atlases to provide complementary coverage of cortical and subcortical regions. The 1035-parcel resolution was adopted to allow for a more granular characterization of lesion-induced disconnections and maximize connectivity mapping accuracy to support individualized patient-level analysis.

The RSN analysis was based on predefined resting-state network regions from the abovementioned atlases. Parcels were delineated based on the resting-state assignments, and each parcel was intersected with the lesion mask to assess the involvement of the corresponding networks. Lesion volumes were calculated for all timepoints, and anatomical lesion locations were matched to Schaefer [56] and (AAL) [57] atlas regions, yielding per-region and per-network quantification.

White matter fiber tracking was performed using the HCP-842 fiber orientation template, a population-averaged diffusion template derived from 842 healthy individuals [58]. Structural connections between parcels were defined by streamlines (white matter fiber pathways) that ended in both parcels. To compute patient-specific structural connectivity matrices at each timepoint, we utilized the LQT [22]. LQT is an open-source MATLAB-based software package designed to quantify the structural impact of focal brain lesions using an atlas-based approach. This method estimates white matter disconnection severity by embedding each patient’s lesion masks into the high-resolution HCP-842 tractography atlas. The reference atlas is a validated template for population-level structural connectome [58]. For every parcel pair, the toolkit identifies the subset of streamlines from the reference template that intersect the spatial lesion volume of each patient’s lesion mask. The proportion of pathways connecting two regions that are intercepted by the lesion represent a “disconnection fraction” and a corresponding “spared fraction”. By accounting for three-dimensional white matter tract trajectories, this approach can estimate disconnections resulting from damage at any point along a tract, providing a biologically informed measure of structural integrity [22]. To compute the weighted structural connectivity matrices post-stroke, we adjusted the healthy atlas streamline counts for each patient based on their specific spared fraction at the three timepoints. Specifically, we computed weighted connectivity as:

weighted_patient(i,j,t) = healthy_weight(i,j) × spared_fraction(i,j,t))

Where:

- healthy_weight(i,j): Represents the end-to-end streamline counts between parcels *i* and *j* as defined by the HCP-842 healthy template.
- spared_fraction(i,j,t)): Represents the proportion of white matter streamlines connecting parcels *i* and *j* that did not intersect with the patient’s lesion at time *t*, calculated as 1 – disconnection_fraction.
- weighted_patient(i,j,t): Is the resulting estimated streamline count for the patient, reflecting the structural integrity of the tract post-stroke.

Binary structural connectivity matrices were further computed by thresholding the weighted structural connectivity matrices, where values > 0 were set to 1 (existing connection) and values = 0 indicated no connection. Existing connections include at least one spared streamline from the HCP-842 atlas, given that the minimum non-zero value was 1 in all weighted connectivity matrices. No connections include connections whose streamlines are fully intersected by the lesion at a given timepoint or if there are no existing streamlines in the healthy atlas, ensuring consistency between the reference connectome and patient-specific matrices.

#### Longitudinal white matter connectivity analysis

To assess how stroke lesions affected network-level connectivity, we first calculated within-network and between-network vulnerabilities, as how much connectivity was lost for each patient and timepoint compared to the healthy atlas using the weighted structural connectivity matrices. Within-network vulnerability was calculated as the summation of connectivity losses between parcels within the same RSN, and between-network vulnerability was calculated as the summation of connectivity losses between parcels in one network and parcels in other networks. The raw connectivity losses were then normalized by the total healthy connectivity to express the RSN vulnerability as a percentage.

We tracked three types of white matter connectivity changes across the three timepoints: between S1 to S2 (early-stage), between S2 to S3 (late-stage), and between S1 to S3 (overall recovery). Disconnections include connections that were present at the earlier timepoint but absent at the later timepoint, identified as lost connections. Re-emerging connections include connections that were absent at the earlier timepoint but present at the later timepoint, identified as gained connections. Over-connections include existing connections (weight > 0 at both timepoints) showing > 10% relative increase in weighted connectivity strength between the timepoints, calculated as ((weight_follow-up – weight_baseline)/weight_baseline) ×100. These three categories are mutually exclusive by construction. To contextualize how much of the discrete connectivity change may be attributable to lesion mask evolution, we computed dice similarity coefficient (DSC) across sessions for individual patients. The values are reported in Supplementary Table 1, right panel. Each edge is classified into exactly one category based on the zero/non-zero status of its weights at the two timepoints being compared. The 10% threshold was selected to identify notable changes in connectivity strength, aligned with prior work showing thresholds at or above 10% represent a conservative criterion that preserves biologically plausible connections at both individual and group level analyses [59]. The threshold was further applied uniformly across patients, as the connectivity changes were quantified relative to each patient’s own matrices at each timepoint, accommodating individual variability in lesion characteristics. Additionally, the differences between the number of re-emerging connections and disconnections were noted for each RSN across the timepoints to assess connectivity gains and losses in recovery at the RSN level.

To further characterize how individual connections (edges) change over time, we performed descriptive edge-level analysis across all unique parcel pairs excluding self-connections for each patient across the three timepoints. While global and RSN-level analyses quantify aggregate connectivity loss and recovery, edge-level analysis identifies which specific connections drive these changes. The same three mutually exclusive categories were applied. For re-emerging connections, we recorded the absolute connectivity strength gained, and for disconnections, the absolute connectivity strength lost. For persistent connections (weight > 0 at both timepoints), we calculated the relative percentage change from baseline. We classified persistent connections showing > 10% relative change (positive or negative) from baseline as showing substantial increases or decreases. To address potential inflation of percentage change estimates when baseline connectivity is near zero, we applied a minimum absolute threshold defined as the 5^th^ percentile of all non-zero baseline edge weights within each transition. Persistent edges with a baseline weight below this floor were excluded from the percentage-change analysis. For each RSN, we quantified involvement in connectivity reorganization by counting connections that showed substantial increases or decreases and calculated the net change as the number of increased connections minus decreased connections. It is important to note that because we analyzed individual patients longitudinally without replication across multiple subjects, this analysis was descriptive and precluded statistical hypothesis testing. The 10% threshold served as a data-driven criterion to quantify the magnitude of observed changes relative to baseline within each patient and aimed to characterize individual patient trajectories and network-level reorganization patterns.

To assess the robustness of findings to threshold selection, a sensitivity analysis was conducted at thresholds of 5% and 20%, alongside the primary 10% threshold. For each threshold, the minimum absolute floor was recomputed from the 5^th^ percentile of non-zero baseline edge weights and applied consistently before percentage change was calculated. Lastly, because connectivity values are derived from a healthy-subject tractography template weighted by spared white matter proportions, the respective differences in connectivity strengths reflect a proportional sparing of atlas-defined pathways and not a direct measurement of strengthened axonal connections.

## Results

Demographic, lesion, and longitudinal clinical characteristics of the patients are summarized in Table 1. Individual trajectories were presented to characterize patient-specific patterns of structural brain reorganization and white matter connectivity changes across post-stroke recovery stages: within 7 days (S1), at ~ 1 month (S2), and at ~ 3 months (S3).

### Lesion evolution

**Fig. 1:**
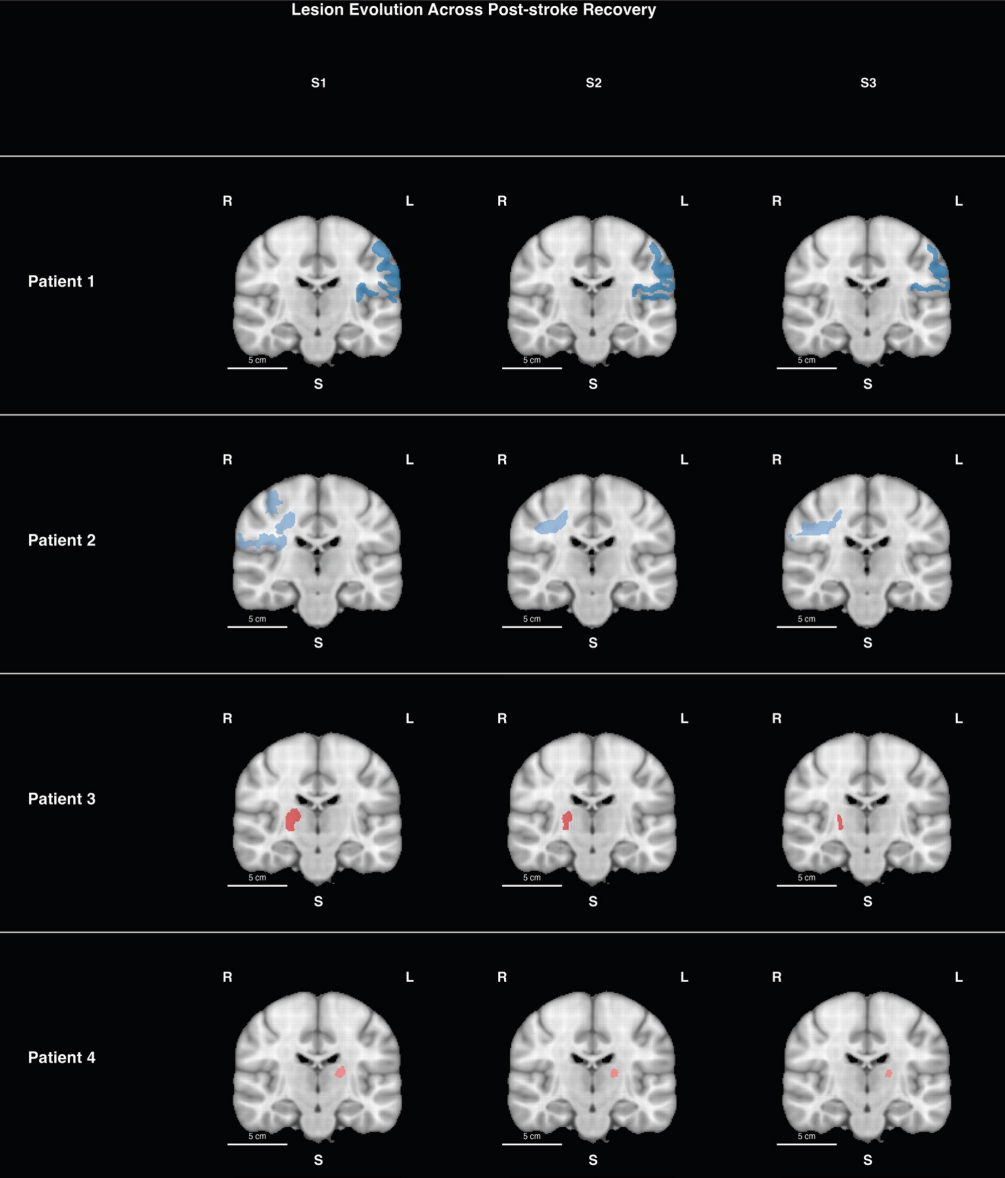
Lesion evolution across post-stroke recovery. Coronal views of lesion masks for four patients displayed on the MNI152 1 mm space at three post-stroke timepoints: S1 – within 1 week, S2 – ~1 month, S3 – ~3 months. Cortical lesions are shown in color blue and subcortical lesions in color red. Each row represents a different patient, illustrating the spatial extent and temporal evolution of lesion boundaries across recovery

Figure 1. illustrates the spatial extent and temporal evolution of stroke lesions across patients. The two cortical stroke patients (Patients 1 and 2) had larger initial volumes (21,428 mm^3^ and 18,141 mm^3^, respectively), compared to the two subcortical stroke patients (Patients 3 and 4; 959 mm^3^ and 312 mm^3^, respectively). The lesion volume progression and percentage recovery at each timepoint for each patient are reported in Table 2.

**Table 2:** Lesion volume evolution and percent change from previous timepoint across post-stroke recovery stages: S1 – within 1 week, S2 – ~1 month, S3 – ~3 months. For percent change, positive values indicate lesion volume reduction; negative values indicate lesion volume increase

		Patient 1	Patient 2	Patient 3	Patient 4
Lesion volume (mm^3^)	S1	21,428	18,141	959	312
	S2	19,198	7,310	441	265
	S3	12,841	8,581	113	141
Percent change from previous timepoint (%)	S1–S2	10%	59.7%	54%	15%
	S2–S3	33.1%	−17.3%	74.3%	46.7%
	S1–S3	40%	52.7%	88.2%	54.8%

### Resting-state network damage and white matter connectivity loss

There were 9 resting-state networks (RSNs) sustaining lesion damage for Patient 1, and 12 RSNs for Patient 2. For both Patients 3 and 4, the only RSN with lesion damage was Caudate Nucleus, values matching the lesion volume evolution. For the cortical stroke patients, the RSNs with the highest baseline lesion damage included SalVentAttnA (Patient 1: 5912 mm^3^, Patient 2: 5641 mm^3^); SomMotB (Patient 1: 5460 mm^3^, Patient 2: 3388 mm^3^) and SomMotA (Patient 1: 1678 mm^3^, Patient 2: 1660 mm^3^). Additionally, two other RSNs with high baseline lesion damage were found as DorsAttnB for Patient 1 (4287 mm^3^), and SalVentAttnB for Patient 2 (2847 mm^3^). The complete RSN-specific lesion volumes are reported in Supplementary Table 2. Lesion volume reductions as percentage by the last recovery stage were found as SalVentAttnA (Patient 1: 24.5%, Patient 2: 70.9%); SomMotB (Patient 1: 60%, Patient 2: 59.7%); SomMotA (Patient 1: 55.6%, Patient 2: 41%) DorsAttnB (Patient 1: 13%), SalVentAttnB (Patient 2: 75.7%).

White matter connectivity loss was quantified for within-RSNs and between-RSNs. The subcortical stroke patients (Patients 3 and 4) exhibited only between-network connectivity losses due to the singular RSN affected by their lesions. Supplementary Figure 1 reports the session evolutions of within- and between-network vulnerability ratios for each RSN for all patients. Below we report the notable changes in white matter connectivity loss for each patient.

For the cortical stroke patients, Patient 1 exhibited the highest baseline within-network connectivity loss in DorsAttnB (40.6% at S1), with limited recovery (36.6% at S3). SomMotB was the second highest RSN with 18.4% connectivity loss at S1, recovering up to 11.6% at S3.

Patient 2 demonstrated pronounced within-network connectivity loss in SomMotA (62.6% at S1), which recovered to 40.4% at S3. DorsAttnB within-network connectivity progressively worsened from 17.8% at S1 to 22.4% at S3.

For between-network connectivity losses, Patient 1 had the highest between-network connectivity loss in SomMotB (23.3% at S1), which initially worsened to 23.7% at S2 before recovering to 18.6% at S3. Similarly, SalVentAttnA’s initial 13.9% between-network connectivity loss at S1 worsened to 16.2% at S2 and returned to 13.8% at S3. Lastly, DorsAttnB showed a similar pattern with worsening from 11.6% (S1) to 13.5% (S2), and to 12.4% (S3).

In Patient 2, SomMotA between-network connectivity loss worsened from 25.7% at S1, to 35.7% at S2, and to 32.1% at S3. SomMotB showed a relatively stable pattern of between-network connectivity loss as 24.9% at S1 to 24.4% at S2, and to 25.7% at S3. Lastly, SalVentAttnA started with 24.2% between-network connectivity loss, that initially recovered to 10.5% at S2 and worsened to 13.6% at S3.

Patient 3 exhibited 18.9% between-network connectivity loss in SomMotA at S1, recovering gradually to 12.3% at S2 and to 8.5% at S3.

Patient 4 exhibited minimal losses. Caudate nucleus initially exhibited 2.8% (S1) between-network connectivity loss, recovering gradually to 2.3% (S2) and to 1.3% (S3).

### Temporal patterns of white matter reorganization

**Fig. 2:**
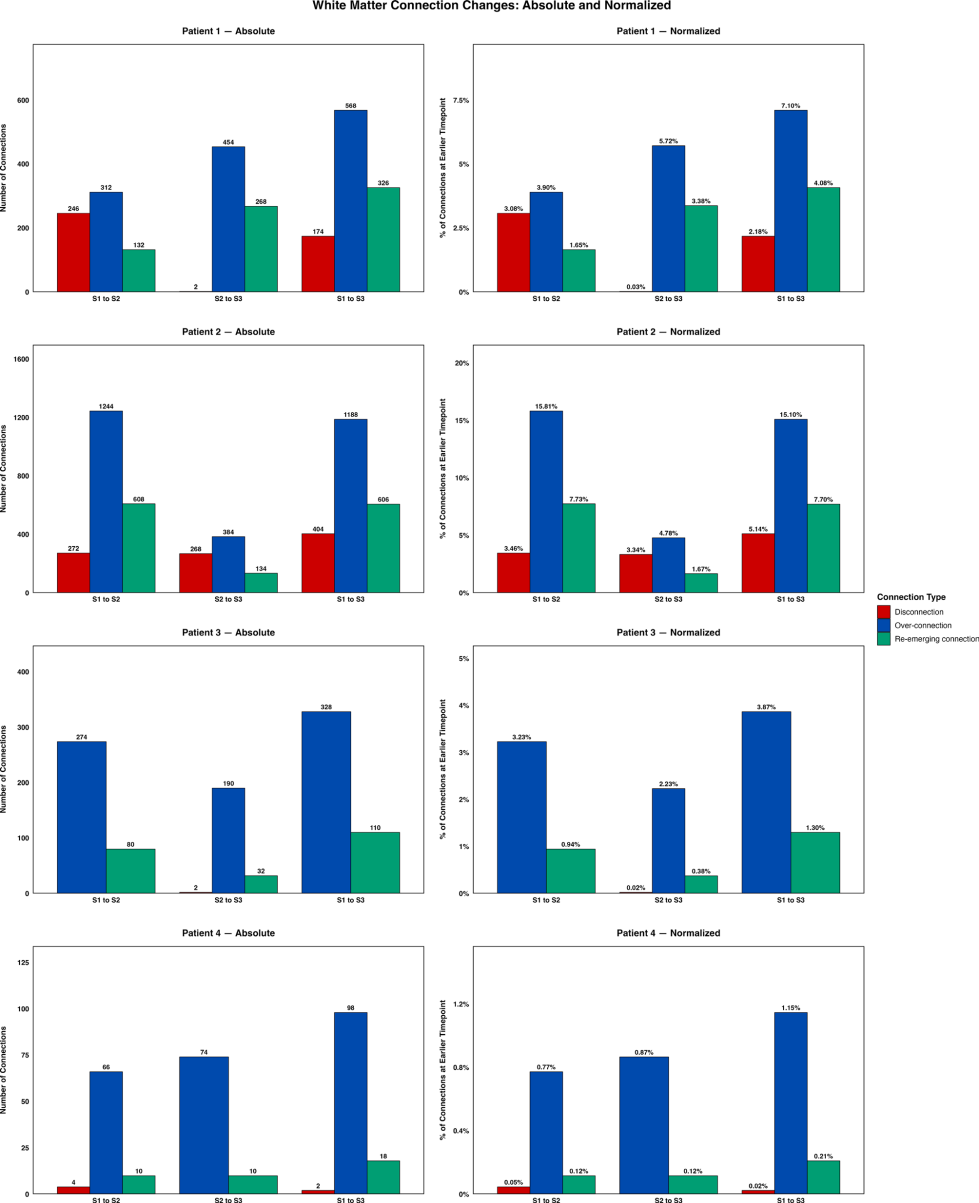
White matter connection changes. For patients 1–4, the total and normalized number of white matter connection changes are shown for three recovery stage transitions: S1 to S2 – within 1 week to ~1 month, S2 to S3 - ~1 to ~3 months, S1 to S3 – within 1 week to ~3 months. The left-side panel shows the absolute counts and the right-side panel shows the normalized counts, calculated as dividing the total number of connections of each type by the total number of connections at the earlier timepoint, expressed as percentages. Disconnections (red) represent connections present in the earlier but absent in the later session, re-emerging connections (green) represent connections absent in earlier but present in the later session, over-connections (blue) represent connections increased their strength by >10% between sessions

We examined the white matter connectivity changes comparing the timepoints across the post-stroke recovery stages: the early-stage (S1 to S2), the late-stage (S2 to S3), and the overall recovery (S1 to S3). Changes were categorized into disconnections, re-emerging connections, and over-connections. Figure 2 illustrates the white matter changes for all patients across recovery stages. The left-side panel shows the absolute counts and the right-side panel shows the normalized counts of connections.

The four patients demonstrated distinct temporal patterns. Patient 1 exhibited early-stage disconnections exceeding re-emerging connections (246 vs. 132), followed by late-stage recovery with minimal disconnections and substantial re-emerging connections (2 vs. 268). Patient 2 demonstrated the opposite pattern, early-stage gains (608 re-emerging vs. 272 disconnections), followed by late-stage losses (134 re-emerging vs. 268 disconnections). Patient 3 showed continuous gains with no early-stage disconnections and 2 late-stage disconnections. Patient 4 maintained stable with minimal changes throughout recovery (≤4 disconnections per stage). Across all patients and recovery stages, the number of over-connections consistently exceeded both those of disconnections and re-emerging connections. The RSN-level connection gains and losses across recovery stage transitions for all patients are shown in Supplementary Figure 2.

### Network-specific contributions to structural reorganization

**Fig. 3:**
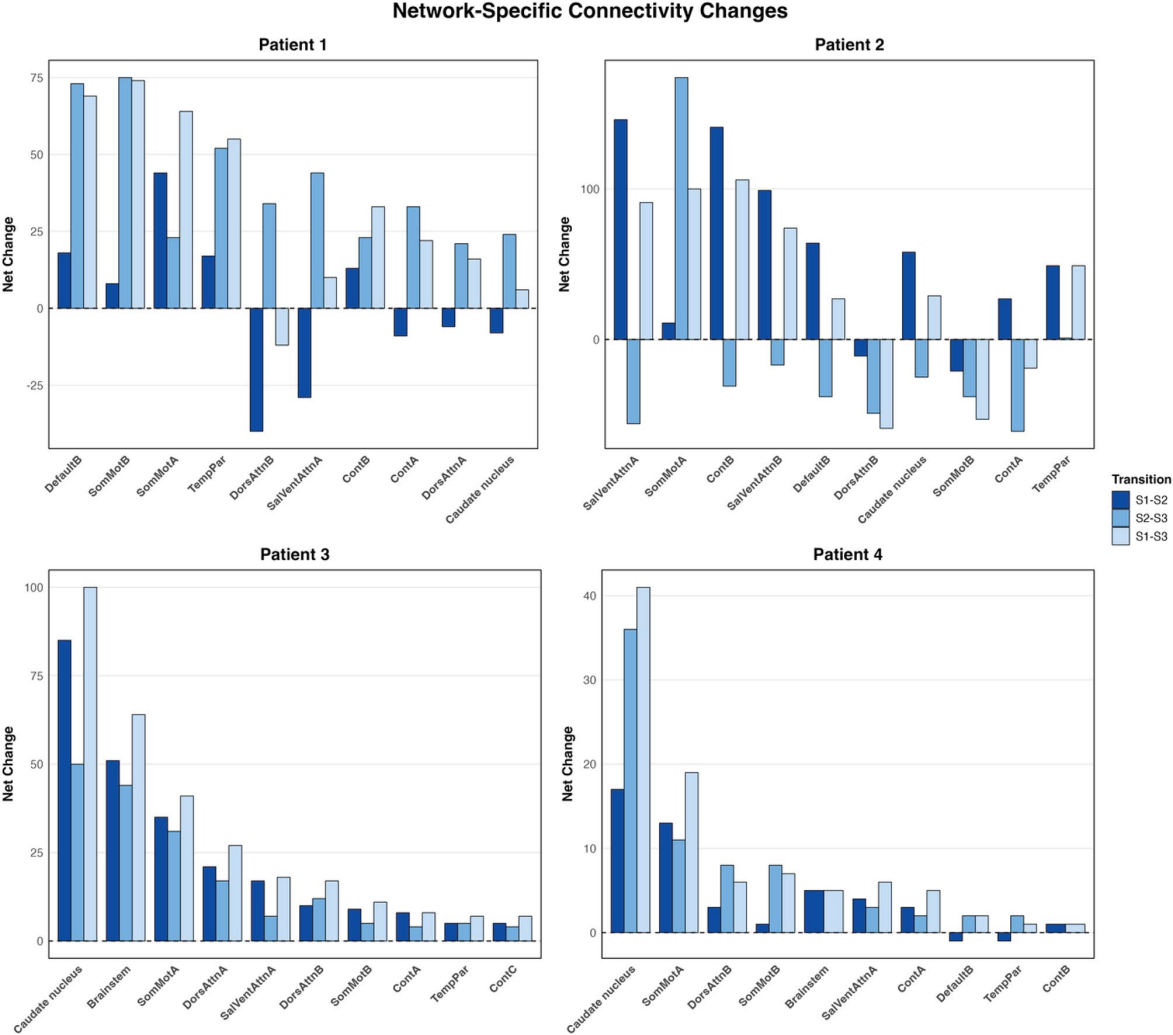
Network-specific substantial connectivity strength changes in white matter connections. Connections are classified as >10% positive or negative change from baseline of each patient. For patients 1–4, the difference between connections with substantial increases and decreases were calculated for each resting-state network across the post-stroke recovery stage transitions: S1–S2 (<1 week to ~1 month), S2–S3 (~1 to ~3 months), and S1–S3 (<1 week to ~3 months). Bars represent the total network involvement, as each edge connects two regions and the changes are attributed to both involved networks

At the edge level, we investigated the substantial changes including increases and decreases in white matter connectivity strength for each RSN. Figure 3. illustrates the network-specific net connectivity changes for each patient and RSN. The following RSNs with the most pronounced white matter reorganization are reported: Somatomotor networks (SomMotA, SomMotB), the salience/ventral attention networks (SalVentAttnA, SalVentAttnB), dorsal attention network (DorsAttnB), and the subcortical Caudate Nucleus network. Supplementary Table 3 summarizes the values for RSN-specific substantial increases, decreases and net changes for each patient across recovery stage transitions. Supplementary Figure 3 further shows the top 10 unique network pairs showing the greatest absolute change in overall recovery for each patient.

### Somatomotor networks

In Patient 1, the somatomotor networks were the highest contributors to connectivity strength increases across all recovery transitions. SomMotA contributed to early-stage connectivity increases (net change: +44), maintained throughout the late-stage (net change: +23) and overall recovery (net change: +64). SomMotB showed fewer early-stage increases (net change: +8) that substantially increased in the late-stage (net change: +75) and overall recovery (net change: +74). In Patient 2, SomMotA had initially few connectivity increases (net change: +11) that increased substantially in the late stage (net change: +174). When the baseline and last recovery stage were compared, SomMotA exhibited a high number of connections with connectivity increases (net change: +100). In contrast, SomMotB exhibited connectivity decreases at all stages (early-stage net change: −21, late-stage net change: −38, overall recovery net change: −53).

Both subcortical stroke patients showed consistent positive somatomotor reorganization. Patient 3’s SomMotA exhibited comparable increases in strength across the early-stage (net change: +35), late-stage (net change: +31), and in overall recovery (net change: +41). SomMotB showed connectivity increases as well, but to a lesser extent, across the early-stage (net change: +9), late-stage (net change: +5), and overall recovery (+11). Similarly, Patient 4’s SomMotA showed comparable connectivity strength increases in the early-stage (net change: +13) and the late-stage (net change: +11), sustained in the overall recovery (net change: +19). SomMotB further showed less connectivity increase across the early-stage (net change: +1) that increased slightly in the late-stage (net change: +8) that is maintained in the overall recovery (net change: +7).

**Fig. 4:**
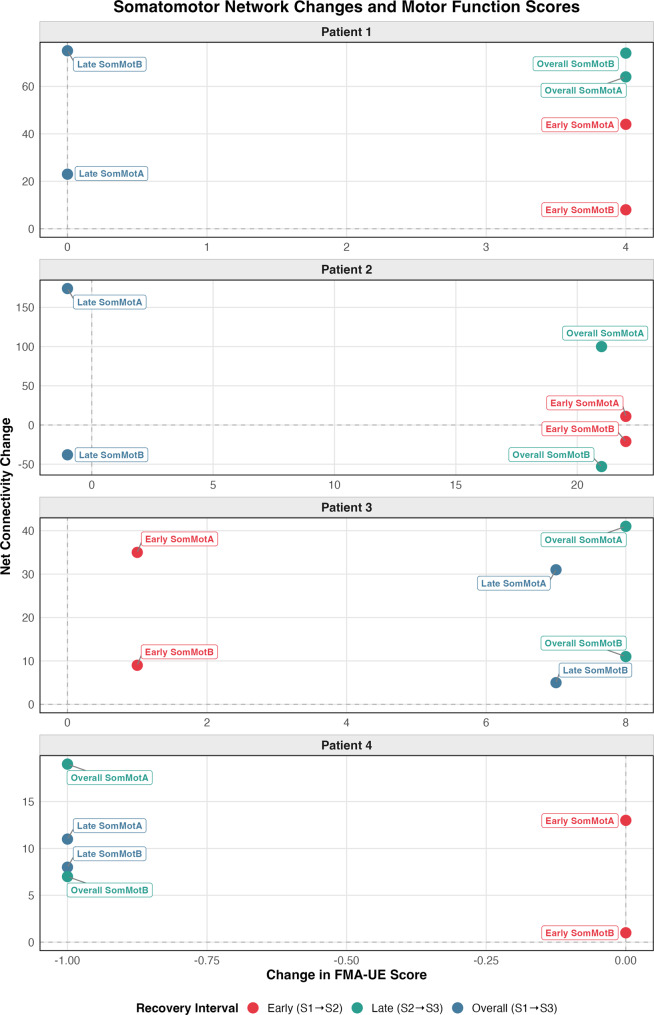
Somatomotor network connectivity changes and motor function scores. For patients 1–4, net changes in substantial connectivity strength (>10%) within SomMotA and SomMotB networks are shown across post-stroke recovery stages: early S1–S2 (<1 week to ~1 month, in red), late S2–S3 (~1 to ~3 months, in blue), and overall S1–S3 (<1 week to ~3 months, in green), alongside corresponding FMA-UE scores. The figure is intended for descriptive comparison and does not imply a quantitative relationship. *SomMota: somatomotor-A, SomMotB: somatomotor-B, FMA-UE: fugl-meyer assessment upper extremity*

To provide an illustrative comparison between network-level connectivity changes and motor function, we present somatomotor network changes alongside FMA-UE scores for each patient in Fig. 4. No formal association is inferred given the small sample size.

### Attention networks: salience/ventral attention networks

Patient 1’s early-stage SalVentAttnA and SalVentAttnB connections showed net connectivity decreases (net change: −29 and −2, respectively), followed by robust late-stage increases (net change: +44 and +16, respectively). In the overall recovery, both salience/ventral attention subnetworks contributed to moderate increases (net change: +10, both). Patient 2 demonstrated the opposite pattern, with both SalVentAttnA and SalVentAttnB showing the highest connectivity increases in the early stage (net change: +146 and +99, respectively), later replaced by decreases in strength in both subnetworks (net change: −56 and −17, respectively). In the overall recovery, both salience/ventral attention subnetworks showed the second highest number of connections with net increases (net change: +91 and +74, respectively) following SomMotA.

Patient 3, SalVentAttnA exhibited early-stage (net change: +17) and late-stage (net change: +7) increases in connectivity strength, reflected in overall recovery as well (net change: +18). SalVentAttnB mirrored this pattern with a slightly greater number of increased connections in the early-stage (net change: +5) than the late-stage (net change: +2) and the overall recovery period (net change: +5). For Patient 4, only SalVentAttnA network connections were found to substantially increase their strength between the timepoints. Few connections showed increases in the early-stage (net change: +4), late-stage (net change: +3) and overall recovery (net change: +6).

### Attention networks: dorsal attention network

Across most of the recovery stages, DorsAttnB network showed net connectivity decreases in cortical stroke patients. In Patient 1, DorsAttnB showed the highest number of connections with substantially decreased strength both in the early stage (net change: −40) and the overall recovery (net change: −12). The late stage exhibited increased connection strength in DorsAttnB (net change: +34). In Patient 2, DorsAttnB showed decreased connectivity strength across the early-stage (net change: −11), late-stage (net change: −49), and the overall recovery (net change: −59).

Patient 3 had a negligible number of connections with substantially decreased strength across the recovery stages. DorsAttnB connections with increased strength were comparable across the early-stage (net change: +10), late-stage (net change: +12), and overall recovery (net change: +17). Patient 4’s DorsAttnB showed few numbers of connections with increased connectivity in the early-stage (net change: +3), the late-stage (net change: +8), and overall recovery (net change: +6).

### Subcortical network: caudate nucleus

For both subcortical stroke patients, Caudate Nucleus exhibited the highest reorganization across all recovery stages. Patient 3 had no connections in caudate nucleus with reduced connectivity strength at any timepoint transition. The increased connections were higher in the early-stage (net change: +85) compared to the late-stage (net change: +50). The overall recovery (net change: +100) exhibited the highest number of connections with increased strength. Patient 4 showed both increased and decreased connectivity strength for caudate nucleus, and the late-stage (net change: +36) included a greater number of connections with increased strength than the early-stage (net change: +17). Similar to Patient 3, the overall recovery (net change: +41) had the highest number of increased connections in Patient 4.

### Sensitivity of edge-level findings to threshold selection

The sensitivity analysis results at 5% 10% 20% thresholds including total substantial change, direction of change, and network involvement are reported in Supplementary Tables 4 and 5. Across all patients and timepoint transitions, the 5^th^ percentile of non-zero baseline edge weights was 1 for each matrix, and no persistent edges were excluded by the minimum absolute floor. The direction and magnitude of connectivity change, along with network stabilities were preserved across thresholds, indicating that the patterns reported for each patient are threshold independent.

## Discussion

This longitudinal case series leveraged ultra-high field 7T MRI and parcel-level connectome analysis to characterize structural network reorganization in four first-time ischemic stroke patients with upper-extremity motor impairments within 1 week, at ~ 1 month and, at ~ 3 months post-stroke. In our sample, the findings highlighted that an increase in estimated connectivity strength of preserved white matter connections emerged as a common mechanism in post-stroke structural network reorganization. Notably, each patient exhibited temporally different patterns in which structural network reorganization was most pronounced. At the network-level, the somatomotor and salience/ventral attention networks facilitated positive adaptive network reorganization through increased connectivity strength. In contrast, the dorsal attention network exhibited persistent decreased connectivity strength in cortical strokes. Below, we summarize key findings for each patient, discuss overarching patterns and network-specific contributions to structural reorganization in this sample.

### Temporally distinct structural reorganization patterns

#### Patient 1: late-dominant, Patient 2: early-dominant, Patient 3: continuous, Patient 4: stable

Our first research question focused on the temporal differences in structural network reorganization in individual patients, and we found that the four patients demonstrated markedly different recovery trajectories. Patient 1 with a cortical lesion showed gradual lesion volume reduction, with the most prominent change in lesion size occurring between ~1 to ~ 3 months. Accordingly, white matter reorganization was found to be most robust at this later stage in recovery. The white matter tracts that were pronouncedly disconnected at the early stage recovered drastically between ~1 to ~ 3 months, with steady improvement of both newly emerging connections and existing connections increasing their estimated connectivity strength. The ~1-month further marked the improvement on the clinical status of Patient 1, as they reached full functional independence (BI:85/100 to 100/100), accompanied by an improved NIHSS score (NIHSS: 3/42 to 2/42), both sustained at ~ 3 months (BI: 100/100, NIHSS: 2/42) (Supplementary Figure 4, first row). Previous literature suggests that one-month timepoint is characterized by a prolonged decrease in growth-inhibitory molecules in the lesioned hemisphere. During this period, axonal sprouting begins, but the novel connections would mature around one month and sustain a stable presence afterwards [37]. These stabilization-type changes could help explain Patient 1’s robust network changes that occur between ~1 to ~ 3 months and remain present, accompanied by improved clinical status.

In contrast, Patient 2 with a cortical lesion showed a substantial decrease in lesion volume by ~ 1 month, followed by a slight increase at ~ 3 months. This late-stage increase may result from processes such as gliosis or delayed infarct evolution, where complex biochemical and cellular mechanisms could disrupt the balance between the brain’s energy demands and available blood flow supply to surrounding tissue [60, 61]. Correspondingly, positive structural network reorganization, characterized by a greater number of novel connections and increased estimated connectivity strength in existing connections, was more pronounced in the early stage. The transition from ~1 to ~ 3 months was instead marked by stable disconnections with fewer new connections. Coinciding with the structural network changes, Patient 2 showed prominent clinical improvements at ~ 1 month. All the clinical scales showed improvement, NIHSS by 5 points (NIHSS: 6/42 to 1/42), mRS by 1 point (mRS: 3/6 to 2/6), and BI by 35 points (BI:60/100 to 95/100). Accordingly, slight worsening was noted for the NIHSS assessment at ~ 3 months (NIHSS score: 1/42 to 2/42), whereas global disability and independence in activities of daily living remained stable (mRS: 2/6, BI: 95/100) (Supplementary Figure 4, second row). The first month has been indicated as a critical stage with enhanced plasticity [10, 62], and early-stage recovery could be attributed to edema and inflammation resolution [63]. The decline in adaptive white matter changes after ~1 month seen in Patient 2’s neural network could be potentially due to mechanisms such as secondary degeneration of the axons and neural degeneration within the remote areas [64, 65].

Patient 3 with a subcortical lesion showed a higher reduction in lesion volume in the early period and exhibited continuous network growth throughout post-stroke recovery stages. The few disconnected paths recovered fully by ~ 3 months, along with sustained novel connections and over-connections at all recovery stage transitions, overall promoting continuous positive reorganization. In terms of clinical status, Patient 3 gradually achieved full functional independence (BI: 50/100 to 65/100 by ~ 1 month, to 100/100 by ~ 3 months). In contrast, both global disability and stroke severity scores showed improvements at the ~3 month assessment, with remaining slight disability being noted (mRS: 4/6 both within 1 week and ~1 month, 2/6 at ~ 3 months; NIHSS: 5/42 within 1 week, 6/42 at ~ 1 month, 3/42 at ~ 3 months) (Supplementary Figure 4, third row).

Lastly, Patient 4 with a subcortical lesion demonstrated substantial reduction in lesion volume at ~ 1 to ~3 months. White matter connection changes remained steady throughout recovery, with a similar number of new connections and over-connections at the early and late stages, and negligible disconnected paths. Aligned with the structural network changes, Patient 4’s clinical status showed maintained functional independence (BI: 100/100 at all timepoints) and ability to perform global activity without significant disability (mRS: 2/6 both within 1 week and ~1 month, 1/6 at ~ 3 months), along with full resolution of stroke symptoms by ~ 3 months (NIHSS: 1/42 both within 1 week and at ~ 1 month, 0/42 at ~ 3 months) (Supplementary Figure 4, last row). A recent study examining subcortical stroke patients revealed that both lesion site and corticospinal tract fiber integrity were linked to different structural reorganization patterns, distinguishing partially and fully recovered patients [66]. The gradual network changes may reflect sustained adaptation and dynamic structural responses not limited to the early stages. Potentially, the extent of structural changes may scale with the degree of lesion-induced disruption, and that the spared connections can support function without extensive structural reorganization.

Nonetheless, some of the observed differences across reorganization patterns likely reflect the anatomical distinction in stroke locations in addition to individual variability. Cortical and subcortical strokes engage distinct structural substrates and subsequent network adaptations [67–70]. Direct damage to the cortex and long cortico-cortical fibers may lead to stronger topological changes in the network [68, 71, 72]. Subcortical lesions do not directly damage cortical nodes but can lead to complex and widespread alterations by disrupting projection pathways (e.g. corticospinal tract, cortico-striatal pathways), and lead to structural changes in cortical regions indirectly connected to the lesion site [66, 73–75]. Alterations in the corticospinal tract integrity and cortico-cortical paths have been found to influence both motor outcomes and recovery potential [76, 77]. In line with this, the two cortical stroke patients (Patients 1 and 2) in our sample had larger lesion volumes affecting directly the somatomotor regions and broader RSN damage, whereas the two subcortical stroke patients (Patients 3 and 5) with damage contained to the caudate nucleus exhibited widespread network alterations affecting somatomotor system connectivity. Thus, rather than purely individual variability, lesion location and size may further represent structural determinants of network reorganization. Given our small sample size and exploratory study design, current observations are hypothesis-generating. Network-level interpretations, including differential contributions of specific resting-state networks should therefore be considered preliminary and descriptive, rather than generalizable.

### Common mechanisms driving adaptive structural network reorganization

The second research question sought to answer if there were common mechanisms in structural network recovery independent of the differences in time and lesion characteristics. In our sample, for all patients and across all recovery stages, we found that white matter reorganization was dominated by an increase in estimated connectivity strength, to a higher degree than those of both new connections and disconnections. Supporting this finding, all patients had substantially more connections with increased strength than decreased strength in the overall recovery stage (<1 week to ~ 3 months). White matter, composed of axonal bundles surrounded by myelin sheaths, has long been recognized as especially vulnerable to ischemic damage [50]. In particular, stroke could affect oligodendrocytes, which play a role in myelin synthesis, contributing to neurodegeneration and demyelination [78]. At the same time, mechanisms such as oligodendrogenesis and glial modulation may occur to promote the repair of myelin structures [79, 80]. The current findings imply that the brain’s response to stroke may involve reinforcing surviving structural pathways over replacing lost connections. Recovery may thus depend more on optimizing the remaining neural network architecture, where surviving connections are prioritized through mechanisms such as increased myelination, which may occur more readily than new synapse formation in the adult brain.

### Structural reorganization along the distributed large-scale networks

Our last research question concerned the longitudinal contribution of pre-defined functional resting state network regions to structural reorganization. Given that all four patients presented upper-extremity motor symptoms, the somatomotor networks were found to exhibit pronounced white matter connectivity changes as hypothesized. Additionally, the network-level analysis revealed that the attention subnetworks differentially contributed to structural reorganization in our sample. The caudate nucleus network regions were further highlighted for the subcortical stroke patients.

### Somatomotor networks: robust network changes

Upper-extremity motor symptoms are highly common after stroke [23, 24] and motor function improvement is suggested to be influenced by motor network reorganization following the acute stage [81, 82].

Patient 1 demonstrated initial vulnerability followed by robust adaptive reorganization across recovery stages. The somatomotor network showed around 24% between-network connectivity loss acutely, which stabilized by ~ 1 month. This pattern is consistent with previous studies showing initial reduction of inter-network functional connectivity between the primary motor cortex and distant brain regions, followed by recovery [83, 84]. This trajectory may reflect early Wallerian degeneration of distal axonal and myelin sheath in the corticospinal tract [85], followed by remyelination and repair mechanisms occurring several weeks after the insult [79]. These repair processes may establish new connections or strengthen existing ones potentially to compensate for the damage to central motor pathway nodes [16, 86]. Supporting this interpretation, substantial connectivity increases emerged both within and between the two somatomotor subnetworks, SomMotA and SomMotB during the overall recovery stage, corresponding with a full upper-extremity motor score (FMA-UE 66/66) by ~ 1 month.

Patient 2 presented a more complex pattern, as the initial between-network connectivity loss (25% at one week) worsened to 35% by ~ 1 month, and remained at 32% by ~ 3 months. This aligns with studies associating greater secondary white matter tract degradation between two weeks to three months post-stroke with worse functional outcomes [87]. Correspondingly, Patient 2’s motor scores improved substantially in early recovery (FMA-UE: 35 to 57/66) but stagnated by ~ 3 months (FMA-UE: 56/66). This suggests that secondary axonal damage may have impaired Patient 2’s somatomotor network reorganization. Notably, the two subnetworks showed divergent trajectories for Patient 2. SomMotA exhibited numerous new connections between ~1 and ~3 months, with substantially increased within-network connectivity strength, reflecting positive adaptive reorganization. In contrast, connectivity between the two subnetworks, and within SomMotB, showed the greatest decreases. This pattern may reflect a compensatory strengthening of local motor circuits when balance or integration between sensorimotor subnetworks is disrupted. While preserved corticocortical or corticothalamic tracts support somatomotor functional connectivity and motor outcomes in stroke patients [88], several mechanisms may contribute to maladaptive plasticity [38]. SomMotB regions’ progressive connectivity loss and decreased strength might result from direct structural damage to critical nodes and secondary diaschisis effects disrupting excitatory-inhibitory balance [33, 89]. Additionally, hyper-reliance on the unaffected side to compensate for functional impairment might have impacted the ipsilesional motor connection reorganization and increase the number of axo-spinous synapses [90], potentially contributing to limited adaptive compensatory reorganization.

The two subcortical stroke patients demonstrated similar positive somatomotor network reorganization patterns, with initially high between-network connectivity loss proportionally recovering across early and late stages. Previous research has shown high functional reorganization of subcortical and motor networks from acute to chronic stages post-stroke, correlating with the structural reorganization during the first one to two weeks [91]. The caudate nucleus maintains extensive cortical connections through large-scale neural networks [92, 93], involving multiple crossing fiber bundles [94]. Consistent with these, both subcortical stroke patients showed substantial increases in somatomotor - caudate nucleus connectivity strength across stages, with caudate nucleus exhibiting most pronounced white matter changes. Motor function recovery followed the general pattern for each patient, Patient 3 showed gradual improvement and reached full function by ~ 3 months, (FMA-UE: 58 to 59 to 66/66), while Patient 4 maintained stable and high scores (FMA-UE: 66 to 66 to 65/66) throughout recovery.

### Attention networks: differential contributions to network changes

The attention subnetworks emerged as critically affected systems in our sample. The dorsal attention network exhibited limited contributions to adaptive network changes for the two cortical stroke patients, while the salience/ventral attention network demonstrated greater contributions for potential compensatory reorganization.

### Dorsal attention network: limited contributions in cortical strokes

The dorsal attention network’s high-order functional roles in top-down attention control and selective information relay to salience/ventral attention network [95, 96] rely on precise long-range connections between higher order areas [97]. Stroke lesions may disrupt the white matter tracts supporting communication within and between attention networks, such as the superior longitudinal fasciculus [98], as well as central hub areas that structurally connect these networks [99].

Patient 1 showed the greatest within-network connectivity loss (~40% acutely, ~36% chronically), with the disconnections remaining high in the overall recovery and poor between-network recovery. The dorsal attention network was the top contributor to the decreased connection strength, particularly in dorsal attention - somatomotor connections during early recovery. Patient 2 demonstrated progressive deterioration in both within- and between-network dorsal attention connectivity through the chronic stage, with a persistent gradual increase in disconnections. Such persistent dysfunction is consistent with the observations that regions of the dorsal attention network can remain diffusely disrupted and fail to recover in some stroke patients [100]. Other studies reported disrupted connectivity between the somatomotor and dorsal attention networks in stroke patients [101, 102], where behavioral deficits were shown to be domain-specific, with attention deficits linked to dorsal attention network disruption, and motor deficits to motor network disruption [102]. Accordingly, Patient 1’s motor improvement and Patient 2’s incomplete motor recovery corresponded with their somatomotor subnetwork reorganization trajectories. Attention performance, however, was not clinically assessed in this study, limiting interpretation of the dorsal attention network findings to attention deficits. Among the subcortical patients, Patient 3’s dorsal attention network was unique in showing more robust improvement in the late rather than the early recovery. Nevertheless, this network contributed to structural recovery by increasing connectivity with the lesioned caudate nucleus. Patient 4 exhibited similar increases in dorsal attention - caudate nucleus connectivity. These trajectories in our sample are consistent with the reports of higher baseline functional connectivity within the dorsal attention network predicting better motor outcomes [103].

### Salience/ventral attention network: compensatory reorganization

In contrast, the salience/ventral attention network demonstrated more pronounced adaptive changes in our sample. Patient 1 showed profound acute between-network connectivity loss that worsened by 16% at ~ 1 month before recovering to acute levels by ~ 3 months. This trajectory was accompanied by substantially increased connectivity between ~1 and ~3 months, particularly in salience/ventral attention – default mode network connections. This pattern aligns with reported functional disruptions in salience/ventral attention regions one to three weeks post-stroke [104], which may contribute to early-stage worsening before robust reorganization occurs. The reported resilience of salience/ventral attention regions in neurodegenerative conditions [105] may support late-stage stabilization. Patient 2 demonstrated initial improvement (~56%) in between-network salience/ventral attention connectivity from within 1 week to ~ 1 month, which subsequently declined by ~29% from ~1 to ~ 3 months. This was reflected in the substantially more novel salience/ventral attention connections during early recovery, later replaced by dominating disconnections. Notably, the two salience/ventral attention subnetworks increased their connectivity strength in the early stage, contributing to positive structural reorganization. For both subcortical stroke patients, salience/ventral attention - caudate nucleus connectivity contributed to the positive changes across recovery stages.

The salience network has been reported to exhibit the highest temporal flexibility and most spatially diverse dynamic functional communication within the brain, making it a core region for flexible inter-network communication [106]. This configuration allows time-varying communication with multiple regions and networks to support different cognitive tasks [106]. Potentially, this flexibility may enable rapid formation of new connections, help establish efficient, adaptable routes, and help explain the differences seen in early and late recovery phases across patients. The observed reorganization patterns between salience/ventral attention and dorsal attention networks suggest that these networks may rely on long-range communication to varying degrees. This difference could potentially reflect variations in structural connections that hub regions maintain with each network, affecting information transfer accordingly [99]. Overall, while interpretation is constrained by the small sample size, this pattern may suggest that persistent disruption of attention-related networks could influence recovery following cortical stroke. Given the role of dorsal attention network in goal-directed attention and the integration of sensory information with motor planning, reduced engagement of this network may limit the functional relevance of structural reorganization observed in other networks. Further investigation in larger cohorts is essential to validate these preliminary observations.

### Methodological considerations and clinical implications

This work utilized patient-specific 7T MRI data alongside a population-level atlas to provide a granular, longitudinal perspective on post-stroke structural reorganization. We bridged lesion anatomy with connectome-level interpretation to summarize complex lesions within an anatomically grounded reference. Traditional diffusion-based analyses often face challenges in lesioned brains due to artifacts like edema-induced signal loss, crossing-fiber ambiguity, and reconstruction failures [107–111]. There is no gold standard for data collection and processing [111, 112], with limited use at 7T in acute stroke [113], though results are improving [114–116]. Therefore, we used 7T structural MRI images for lesion delineation. To estimate connectome-level lesion disruption, we leveraged the HCP-842 population-averaged atlas which incorporates high-resolution diffusion MRI data from a large healthy adult sample with expert-vetted streamlines [117]. This provided a reliable structural baseline for characterizing what is anatomically conserved in the presence of variable lesion-induced disruption patterns [22]. Parcel- and network-level connectivity were then estimated using the LQT through expected disconnections given a lesion location relative to the reference connectome, which is suggested to be biologically grounded in neural signal transmission mechanisms [22]. This approach ensured consistent quantification across patients and timepoints, but it has specific boundaries. Because the derived connectivity matrices represent estimated pathway integrity, they are connectome approximates of lesion effects and not direct measurements of patients’ own white matter. The observed increases in estimated connectivity strength reflect a higher proportion of spared streamlines as lesions evolve and do not provide direct evidence of microstructural remodeling. The longitudinal changes are likely driven by lesion mask evolution, including edema resolution, tissue consolidation, and subsequent changes in MRI characteristics and spatial boundaries [107, 118–121]. To systematically evaluate the connectivity change relationship to lesion mask overlap and volume change, we added Supplementary Figure 5–8. The analyses confirmed that the direction and magnitude of connectivity changes co-varied with lesion evolution. Re-emerging connections tracked lesion shrinkage and boundary contraction, while disconnections were less tied to volume change amount. Greater boundary shifts were linked to higher re-emerging connection ratios, consistent with the interpretation that spatial mask contraction driving streamline uncovering. Nonetheless, while we can infer how lesion evolution alters the estimated integrity of the structural network, genuine microstructural remodeling cannot be claimed without future validation using patient-specific diffusion MRI metrics (e.g. fractional anisotropy, mean diffusivity, fixel-based analysis [122, 123] or myelin-sensitive imaging [124].

We implemented a 1035-parcel resolution, as evidence suggests that resolution is a more critical determinant of connectome representation than the precise parcel border locations [125]. In a stroke context, coarser parcellations risk diluting the signal from smaller lesions by absorbing disruptions into larger regions [125, 126]. Fine-grained approaches improve signal-to-noise ratio and maximize parcel stability [56, 127], and were reported to provide more reliable biomarkers in brain disorders [128]. This granularity enabled us to identify specific edge-level connection pairs in our sample, such as somatomotor-caudate nucleus pathways in subcortical strokes, or dorsal attention – somatomotor pathways in cortical strokes, offering insights beyond global or RSN-level summaries. To mitigate segmentation variability and registration errors associated with high granularity [126, 129], we implemented several quality control measures. Following the gold standard [130–133], lesions were manually segmented by a rater with extensive neuroanatomical expertise in a single setting across all timepoints to minimize intra-rater drift. Delineation was performed on each timepoint’s high-resolution 0.75 mm anatomical images to maximize internal consistency. Lesion registration used a two-step approach combining affine and enantiomorphic normalization, well suited to lesioned brains [134], with visual inspections to ensure accuracy. Despite these rigors, some atlas- or segmentation-dependent artifacts remain possible, making it difficult to disentangle biological remodeling and methodological variation, as observed in the lesion volume fluctuations in Patient 2. Therefore, while lesion evolution provides a coherent mechanistic explanation for the observed connectivity changes within this framework, we cannot fully separate biological processes from methodological influences. Nonetheless, high granularity ensures that even small lesions are sufficiently represented even when atlas does not perfectly align with each individual’s unique anatomy.

We used connectivity biomarkers to provide a framework pointing towards potential areas for clinical investigation in stroke recovery. While scales like NIHSS and FMA-UE provide essential snapshots of impairment, the connectome approach helps investigate the structural why behind recovery trajectories. The findings suggest that parcel-level connectome analysis may provide information beyond conventional imaging markers and clinical scales by capturing network-level structural dynamics that are not reflected in lesion size or early functional scores. Prior diffusion MRI work showed that early-stage white matter changes can predict recovery over the first three months independent from the lesion size and region [46]. Compared to diffusion-based tract analyses which primarily assess microstructural integrity of predefined pathways e.g. the corticospinal tract, the current approach provides a complementary, systems-level perspective by quantifying how lesions impact distributed network architecture and how these effects evolve longitudinally relative to a normative connectome.

The observations within our sample showed that functional gains and structural remodeling do not always unfold in parallel. For instance, Patient 1 reached a full functional score early, while peak structural changes peaked later. Patient 2 showed stable functional scores at one month and three months, but distinct patterns of connection changes were still ongoing. This suggests that clinical outcomes and structural reorganization can follow separate timelines. The observed dissociation between clinical recovery and structural reorganization across patients highlights the potential for connectome-based metrics to explain inter-individual variability that remains unexplained by standard assessments.

Our edge-level analysis further suggests that different functional systems might reorganize at different rates and directions. In this sample, somatomotor and salience/ventral attention networks showed signs of adaptive changes, while dorsal attention network appeared less amenable for positive reorganization particularly in cortical stroke cases. This may be particularly relevant in cases where widespread or indirect network disruptions extend beyond focal tract damage. The observations create a foundation for future large-scale studies to explore if intensive rehabilitation can be timed to match specific network reorganization windows. From a clinical perspective, this framework may offer a basis for identifying patient-specific windows of heightened network plasticity, which could in future studies inform the timing of rehabilitation or guide neuromodulation strategies toward specific network interactions. For example, somatomotor or salience/ventral attention network connectivity may be targeted in patients with preserved motor pathways but limited integrative network engagement. We acknowledge the small sample size and emphasize that this work serves as an exploratory, hypothesis-generating tool that offers incremental value for understanding recovery mechanisms, and upon validation in larger cohorts, informing personalized rehabilitation strategies.

The Food and Drug Administration (FDA) and Conformité Européene (CE) approved 7T MRI for clinical use [135], and our study adds to the growing evidence for the feasibility of multimodal 7T protocols [113, 136, 137]. Approximately one hour scan time was tolerated in acute patients as early as two days post-stroke, consistent with reports of generally acceptable tolerability [138, 139]. Although 7T systems offer superior imaging, they remain largely confined to research centers [140], due to strict safety regulations and implant incompatibility, high costs, and specialized personnel requirements [135, 141, 142]. Current 7T stroke studies mostly focus on carefully selected small cohorts with stable clinical status [136, 137, 143], leaving acute and vulnerable groups underrepresented. Since stroke is highly heterogenous, varying with age, pre-existing neural network architecture, and lesion location, individualized longitudinal tracking is essential. Ultra-high field imaging offers clear advantages including better lesion mapping, precise microvascular details, and high sensitivity to subtle structural changes [136, 137, 144], all of which are relevant to prognosis and monitoring. As new technologies improve technical challenges and safety standards [114, 145, 146], standardizing the underutilized protocols with expanding clinical research [145] will be key to moving 7T imaging into routine clinical practice.

Our connectome framework is reproducible and translatable to conventional 3T systems. Because the Schafer-Yeo parcellations map to established RSNs, network-level observations remain comparable across studies. The analytical pipeline relies on standard, validated tools applicable to 3T systems and clinical protocols to support multi-center validation. However, differences in signal characteristics and lesion delineation accuracy between 7T and 3T systems may influence sensitivity to subtle connectivity changes, and this should be systematically evaluated in future studies. An additional consideration is that the choice of parcellation resolution and subsequent parcel-RSN aggregations can impact the sensitivity to scale-dependent reorganization patterns. Future large-cohort studies should consider multi-resolution approaches spanning both coarse and fine scales to balance detailed spatial characterization with the statistical power needed to generalize these prospective findings.

## Conclusion

This longitudinal case series leveraged ultra-high field 7T MRI and parcel-level connectome analysis to longitudinally characterize post-stroke structural network reorganization in individual patients. Increase in estimated connectivity strength of surviving connections was observed as a common adaptive mechanism across patients in our sample, suggesting that recovery may in some instances, prioritize optimization of remaining pathways over establishing new ones. At the network level, somatomotor and salience/ventral attention networks demonstrated robust adaptive changes, whereas dorsal attention network exhibited a more limited contribution to adaptive network changes particularly in the cortical strokes examined in this study. The timing of peak reorganization varied across patients; from early-dominant to late-dominant patterns, highlighting a potential need for individualized assessment to test the association with the clinical outcomes in larger populations. Collectively, these preliminary observations provide a detailed characterization of longitudinal structural network reorganization post-stroke.

## Electronic supplementary material

Below is the link to the electronic supplementary material.

Supplementary Material 1

## Data Availability

Raw and processed MRI data that have been used are confidential and not openly available due to data sensitivity. Other data will be made available upon reasonable request from the corresponding author. Anonymized data are located in encrypted data storage at Norwegian University of Science and Technology.
