# Supplementary material for: Structural connectome changes as biomarkers of stroke recovery: a longitudinal 7T MRI study
Source: BMC Neurosci. 2026 May 14;27:19. doi: 10.1186/s12868-026-01010-9 (PMC13220373; doi:10.1186/s12868-026-01010-9)
Supplement: Supplementary file 1 — Supplementary Material 1 [file 12868_2026_1010_MOESM1_ESM.pdf]

Structural Connectome Changes as Biomarkers of Stroke Recovery: A Longitudinal 7T MRI Study by Avci-Colak et al.

Supplementary Information

MRI data acquisition

Neuroimaging data were acquired using 7 Tesla MRI scanner (Magnetom Terra, equipped with a Nova Medical Head Coil 1TX/32RX, Siemens Healthcare GmbH, Erlangen, Germany). High-field T1-mapping was done by a whole-brain coverage 3D-MP2RAGE (224 slices; FOV =168 x 240 x 240 mm; GRAPPA acceleration factor =3; isotropic resolution =0.75 ×0.75 × 0.75 mm; TR =4.3 s; TI1/TI2 =0.84/2.37 s; FA1/FA2 =5°/6°; TE =1.99 ms; echo spacing =7.2 ms; bandwidth =250 Hz/Px; partial-fourier =6/8; acquisition time =9.25 min). High-field T2-mapping was done by a 3D-FLAIR (224 slices; FOV =168 x 202.5 x 240 mm; CAIPIRINHA acceleration factor =3; isotropic resolution =0.75 ×0.75 ×0.75 mm; TR =5 s; FA =120°;TE =2.48 s; echo spacing =3.92 ms; bandwidth =651 Hz/Px; acquisition time =6.55 min).

Structural preprocessing and lesion segmentation from previous work (1)

T1-weighted and T2-FLAIR images were defaced using the *pydeface* toolbox (2) for anonymization. MP2RAGE, including the INV2 and UNI T1 images were processed using *presurfer*, a modified SPM-based anatomical preprocessing tool (3). Processing steps included bias correction and normalization of INV2 images (4), multiplication with UNI images to discard salt-and-pepper noise artifacts, followed by bias correction of denoised UNI images and segmentation by SPM *NewSegment*. FLAIR images were processed similarly by SPM bias correction and segmentation and registered to the UNI images using boundary-based registration (5) via UNI-segmented white matter masks. Brain extraction was performed using HD-BET (6), followed by manual removal of sagittal sinus from all images (7). All images were further inspected for the quality of brain extraction.

Lesion registration

Binary lesion masks in native space were dilated using MRtrix3 *maskfilter* command (8). T1 images were affine registered to MNI space (0.7 mm resolution template) using FSL *flirt* (9). The dilated lesion mask was registered to MNI space using *flirt -applyxfm*, and healthy tissue from the contralesional hemisphere was masked using *fslmaths*, flipped back along the x-axis using *fslswapdim*. Finally, the inverse affine transformation was applied using *convert_xfm* and the healthy tissue was registered from MNI space back to native space using FSL *flirt*. In native space, an additional mask of healthy tissue was generated using *fslmaths* to fit the lesion boundary. The original lesion mask was inverted using *fslmaths*. Then, the T1-image was multiplied by the inverted mask to create a T1-pitted image using *fslmaths*. The healthy tissue mask was added to the T1-pitted image, generating the enantiomorphic filled T1 image. Using ANTs (10) symmetric diffeomorphic registration (11) was done as the MNI space with 1 mm isotropic voxel dimensions as the fixed image and enantiomorphic filled T1 image as the moving image. The *antRegistrationSyn* command uses double precision and includes the registrations Euler3D Transform, Affine Transform and finally SyN transformations (12), generating the generic affine matrix, warped and inverse warped images. This approach was chosen to ensure the alignment of anatomical images to the MNI space. Finally, the lesion in native space was registered to MNI space using *antsApplyTransforms* using only the generic affine matrix. This approach was chosen to ensure the alignment for lesion location and preservation of lesion volume in the MNI space, and the warp field was omitted to prevent distortion of lesion volume. The lesion alignment to both T1 image and MNI space was checked

throughout all stages, in addition to normalized mutual information (NMI) metric to ensure accuracy of lesion localization and borders.

White matter tractography software and parameters
White matter fiber tracking was performed using DSI Studio (13)

Supplementary Tables and Figures

	Session	NMI	Patient_Mean_NMI	Deviation_from_Mean
Patient	S1	1,0726	1,0724	0,0002
	S2	1,0702	1,0724	0,0022
	S3	1,0743	1,0724	0,0019
Patient 2	S1	1,0269	1,0282	0,0013
	S2	1,0285	1,0282	0,0003
	S3	1,0292	1,0282	0,001
Patient 3	S1	1,0566	1,0635	0,0069
	S2	1,0665	1,0635	0,003
	S3	1,0675	1,0635	0,004
Patient 4	S1	1,0294	1,0284	0,001
	S2	1,028	1,0284	0,0004
	S3	1,0277	1,0284	0,0007

Patient	Timepoint	DSC
Patient 1	S1 vs S2	0,5915
	S2 vs S3	0,7147
	S1 vs S3	0,506
Patient 2	S1 vs S2	0,2428
	S2 vs S3	0,6157
	S1 vs S3	0,2241
Patient 3	S1 vs S2	0,5756
	S2 vs S3	0,4485
	S1 vs S3	0,2381
Patient 4	S1 vs S2	0,6804
	S2 vs S3	0,6523
	S1 vs S3	0,4669

Supplementary Table 1. Left panel: Normalised mutual information (NMI) values across sessions between patient-specific anatomical image and the Montreal Neurological Institute template. Maximum absolute deviation from the patient's longitudinal mean NMI was computed to assess registration quality. Right panel: Dice similarity coefficient (DSC) values across timepoints for each patient. Higher values indicate lower spatial overap in the proportion of lesion mask boundaries between timepoints.

Patient 1			
**RSN_Name**	**Lesion_Volume_S1**	**Lesion_Volume_S2**	**Lesion_Volume_S3**
SalVentAttnA	5912	5691	4408
SomMotB	5460	5081	2171
DorsAttnB	4387	4782	3810
SomMotA	1678	1011	744
ContA	1192	841	708
SalVentAttnB	1053	945	708
ContB	843	62	131
TempPar	464	553	2
DefaultB	420	232	159
DefaultA	14	0	0
DorsAttnA	5	0	0

Patient 2			
**RSN_Name**	**Lesion_Volume_S1**	**Lesion_Volume_S2**	**Lesion_Volume_S3**
SalVentAttnA	5641	1325	1638
SomMotB	3388	1708	1363
SalVentAttnB	2847	600	690
SomMotA	1660	1026	979
DefaultB	1397	502	765
DorsAttnB	985	1233	1752
ContA	785	626	1157
ContB	717	221	228
Caudate nucleus	514	0	0
LimbicB	193	69	9
DefaultA	11	0	0
TempPar	3	0	0

Patient 3			
**RSN_Name**	**Lesion_Volume_S1**	**Lesion_Volume_S2**	**Lesion_Volume_S3**
Caudate nucleus	959	441	113

Patient 4			
**RSN_Name**	**Lesion_Volume_S1**	**Lesion_Volume_S2**	**Lesion_Volume_S3**
Caudate nucleus	312	265	141

Supplementary Table 2. Resting state network (RSN) regions sustaining lesion damage (mm^3) for Patients 1-4 across post-stroke recovery timepoints: S1 (< 1 week), S2 (at 1 month), and S3 (at 3 months).

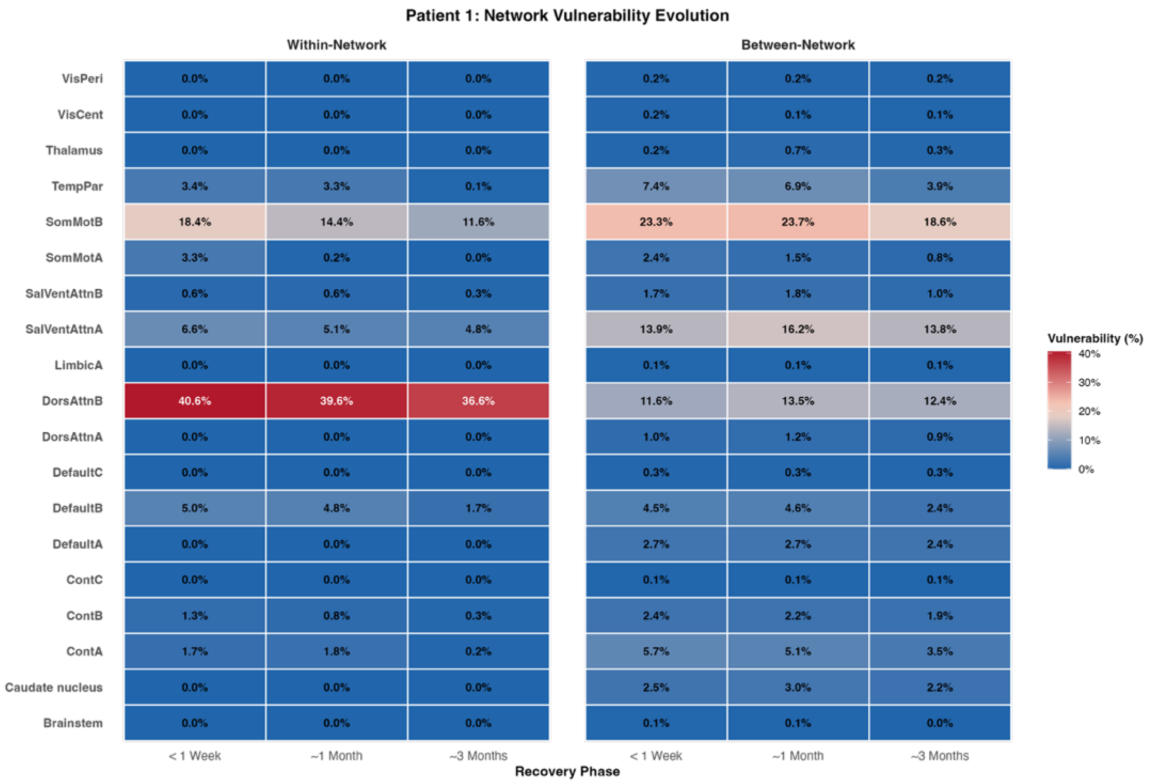
Patient 1: Network Vulnerability Evolution
Within-Network
Between-Network
VisPeri 0.0% 0.0% 0.0% 0.2% 0.2% 0.2%
VisCent 0.0% 0.0% 0.0% 0.2% 0.1% 0.1%
Thalamus 0.0% 0.0% 0.0% 0.2% 0.7% 0.3%
TempPar 3.4% 3.3% 0.1% 7.4% 6.9% 3.9%
SomMotB 18.4% 14.4% 11.6% 23.3% 23.7% 18.6%
SomMotA 3.3% 0.2% 0.0% 2.4% 1.5% 0.8%
SalVentAttnB 0.6% 0.6% 0.3% 1.7% 1.8% 1.0%
SalVentAttnA 6.6% 5.1% 4.8% 13.9% 16.2% 13.8%
LimbicA 0.0% 0.0% 0.0% 0.1% 0.1% 0.1%
DorsAttnB 40.6% 39.6% 36.6% 11.6% 13.5% 12.4%
DorsAttnA 0.0% 0.0% 0.0% 1.0% 1.2% 0.9%
DefaultC 0.0% 0.0% 0.0% 0.3% 0.3% 0.3%
DefaultB 5.0% 4.8% 1.7% 4.5% 4.6% 2.4%
DefaultA 0.0% 0.0% 0.0% 2.7% 2.7% 2.4%
ContC 0.0% 0.0% 0.0% 0.1% 0.1% 0.1%
ContB 1.3% 0.8% 0.3% 2.4% 2.2% 1.9%
ContA 1.7% 1.8% 0.2% 5.7% 5.1% 3.5%
Caudate nucleus 0.0% 0.0% 0.0% 2.5% 3.0% 2.2%
Brainstem 0.0% 0.0% 0.0% 0.1% 0.1% 0.0%
< 1 Week
~1 Month
~3 Months
< 1 Week
~1 Month
~3 Months
Recovery Phase
Vulnerability (%)
40%
30%
20%
10%
0%

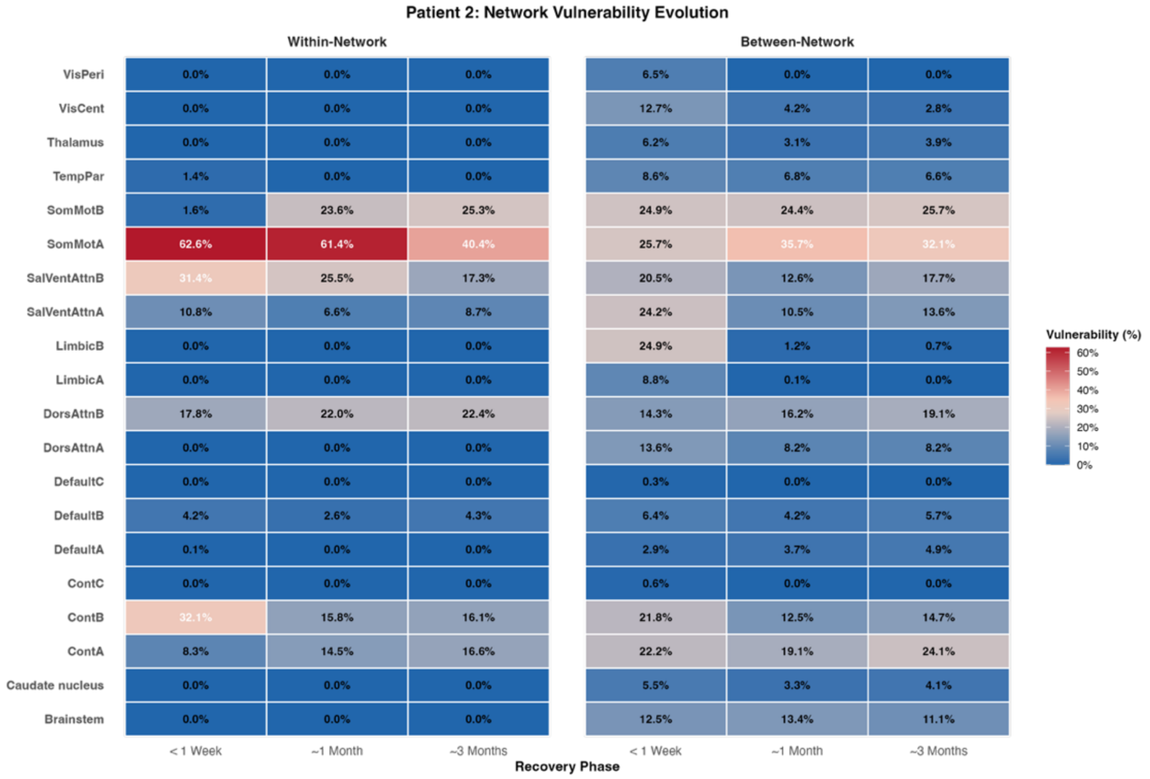
Patient 2: Network Vulnerability Evolution
Within-Network
Between-Network
VisPeri 0.0% 0.0% 0.0% 6.5% 0.0% 0.0%
VisCent 0.0% 0.0% 0.0% 12.7% 4.2% 2.8%
Thalamus 0.0% 0.0% 0.0% 6.2% 3.1% 3.9%
TempPar 1.4% 0.0% 0.0% 8.6% 6.8% 6.6%
SomMotB 1.6% 23.6% 25.3% 24.9% 24.4% 25.7%
SomMotA 62.6% 61.4% 40.4% 25.7% 35.7% 32.1%
SalVentAttnB 31.4% 25.5% 17.3% 20.5% 12.6% 17.7%
SalVentAttnA 10.8% 6.6% 8.7% 24.2% 10.5% 13.6%
LimbicB 0.0% 0.0% 0.0% 24.9% 1.2% 0.7%
LimbicA 0.0% 0.0% 0.0% 8.8% 0.1% 0.0%
DorsAttnB 17.8% 22.0% 22.4% 14.3% 16.2% 19.1%
DorsAttnA 0.0% 0.0% 0.0% 13.6% 8.2% 8.2%
DefaultC 0.0% 0.0% 0.0% 0.3% 0.0% 0.0%
DefaultB 4.2% 2.6% 4.3% 6.4% 4.2% 5.7%
DefaultA 0.1% 0.0% 0.0% 2.9% 3.7% 4.9%
ContC 0.0% 0.0% 0.0% 0.6% 0.0% 0.0%
ContB 32.1% 15.8% 16.1% 21.8% 12.5% 14.7%
ContA 8.3% 14.5% 16.6% 22.2% 19.1% 24.1%
Caudate nucleus 0.0% 0.0% 0.0% 5.5% 3.3% 4.1%
Brainstem 0.0% 0.0% 0.0% 12.5% 13.4% 11.1%
< 1 Week
~1 Month
~3 Months
< 1 Week
~1 Month
~3 Months
Recovery Phase
Vulnerability (%)
60%
50%
40%
30%
20%
10%
0%

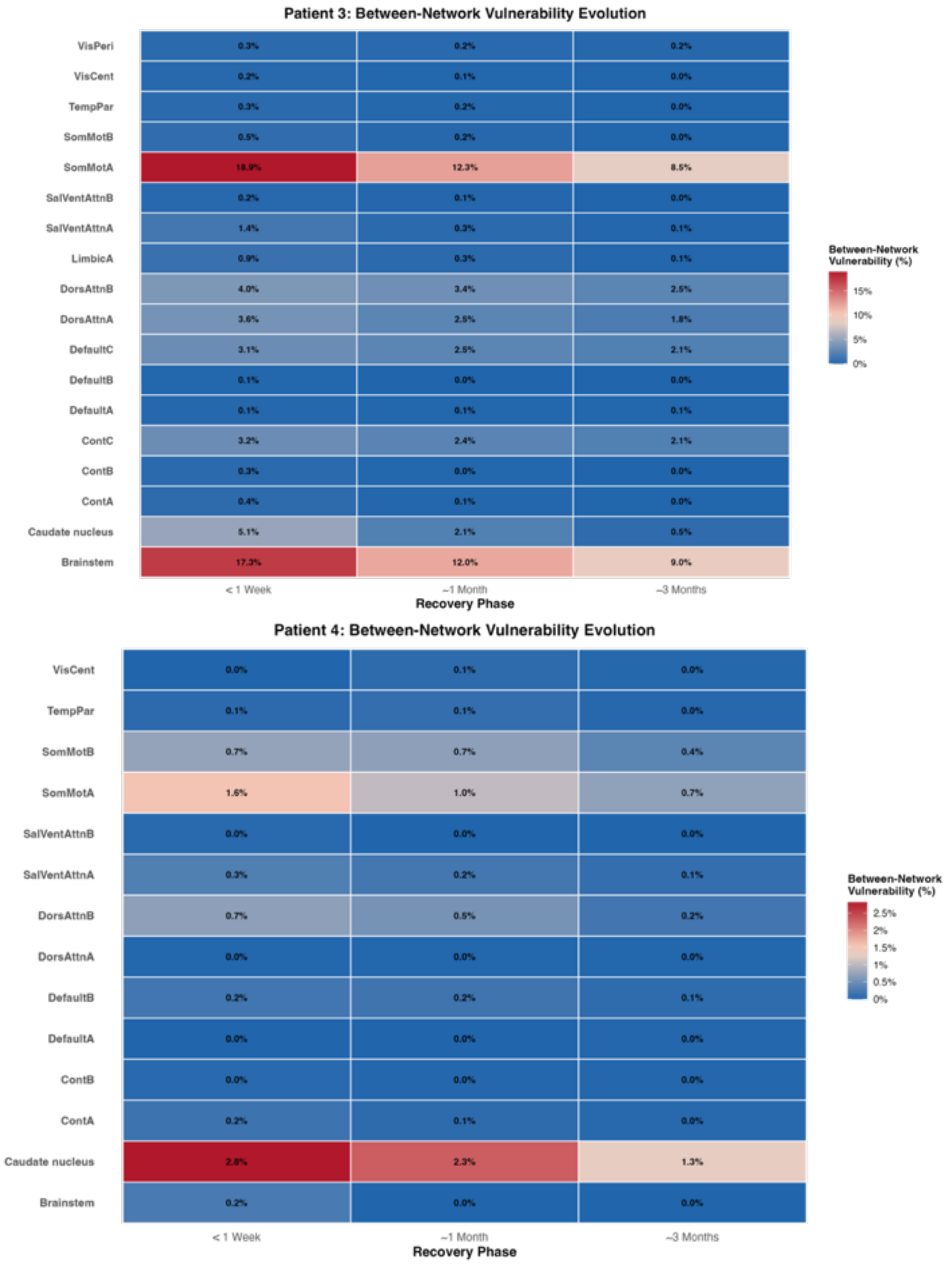

Supplementary Figure 1. Within- and between-network RSN vulnerability ratios for Patients 1-4 across post-stroke recovery stages: < 1 week, at 1 month, at 3 months. Within-network vulnerability is calculated as sum of weighted connectivity decreases among parcels in the same RSN and between-network vulnerability as sum of weighted connectivity decrease between parcels in one RSN and the other RSNs, normalized as percentage of total healthy weighted connectivity.

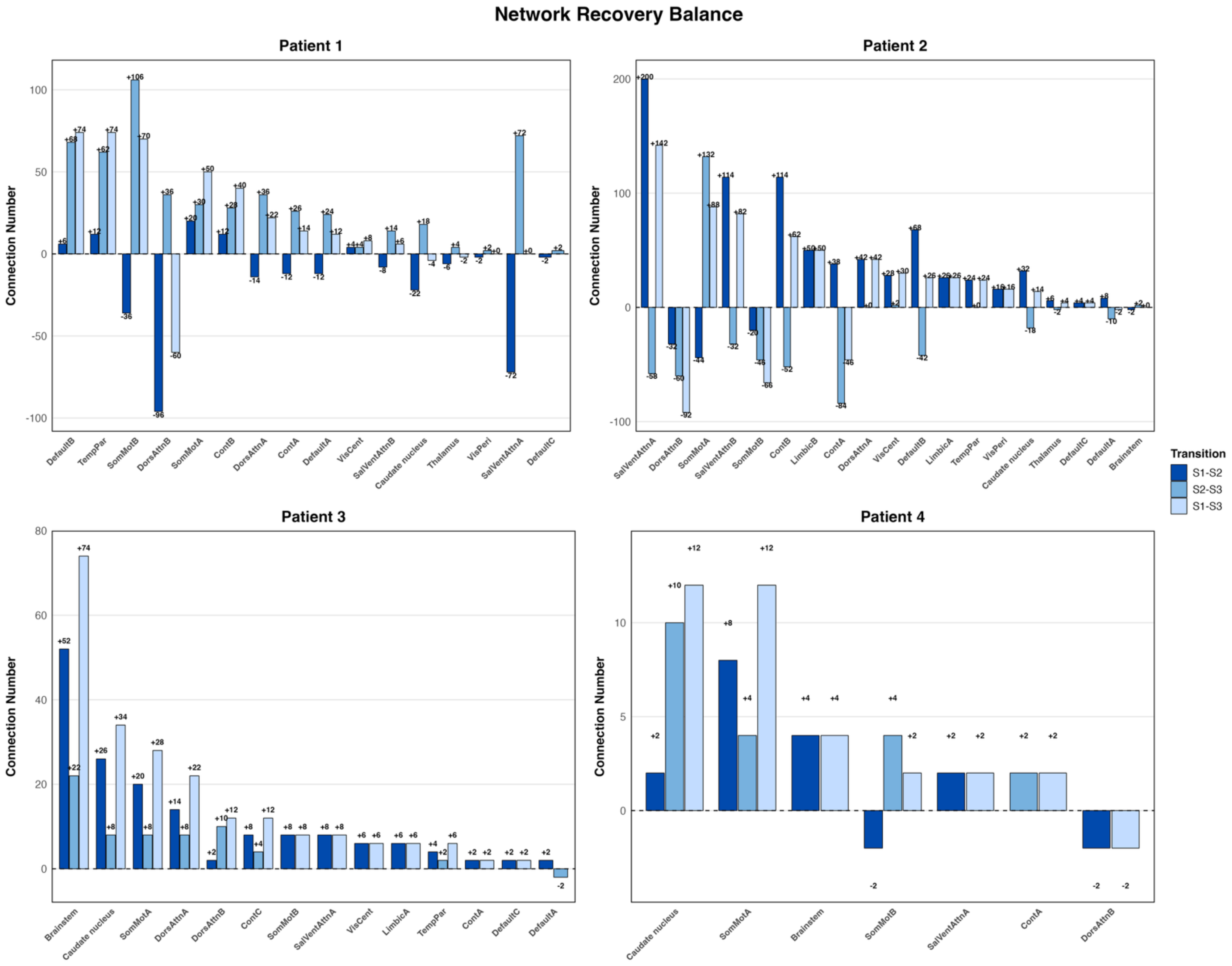

Supplementary Figure 2. RSN-level network recovery for Patients 1-4. Positive values indicate connection gains (more number of re-emerging connections than disconnections) and negative values indicate connection losses (more number of disconnections than re-emerging connections). Post-stroke recovery stages are S1: < 1 week, S2: at 1 month, and S3: at 3 months.

S1-S2				S2-S3				S1-S3			
**Patient 1**											
**RSN**	**Increasing**	**Decreasing**	**Net_Change**	**RSN**	**Increasing**	**Decreasing**	**Net_Change**	**RSN**	**Increasing**	**Decreasing**	**Net_Change**
SomMotA	52	8	44	SomMotB	75	0	75	SomMotB	97	23	74
DefaultB	44	26	18	DefaultB	74	1	73	DefaultB	79	10	69
TempPar	40	23	17	TempPar	55	3	52	SomMotA	67	3	64
ContB	23	10	13	SalVentAttnA	48	4	44	TempPar	73	18	55
SomMotB	51	43	8	DorsAttnB	34	0	34	ContB	39	6	33
VisCent	3	0	3	ContA	33	0	33	ContA	36	14	22
Brainstem	1	0	1	ContB	25	2	23	DorsAttnA	29	13	16
VisPeri	0	1	-1	DorsAttnA	23	2	21	SalVentAttnA	50	40	10
DefaultC	0	1	-1	Caudate nucleu	24	0	24	DefaultA	18	8	10
SalVentAttnB	8	10	-2	SomMotA	23	0	23	SalVentAttnB	16	6	10
DefaultA	9	12	-3	DefaultA	17	0	17	Caudate nucleu	17	11	6
Thalamus	0	3	-3	SalVentAttnB	16	0	16	VisCent	5	0	5
DorsAttnA	11	17	-6	VisCent	2	0	2	Brainstem	1	0	1
Caudate nucleu	9	17	-8	Thalamus	2	0	2	VisPeri	1	1	0
ContA	18	27	-9	VisPeri	1	0	1	Thalamus	0	1	-1
SalVentAttnA	23	52	-29	DefaultC	1	0	1	DorsAttnB	40	52	-12
DorsAttnB	20	60	-40	Brainstem	1	0	1				
**Patient 2**											
**RSN**	**Increasing**	**Decreasing**	**Net_Change**	**RSN**	**Increasing**	**Decreasing**	**Net_Change**	**RSN**	**Increasing**	**Decreasing**	**Net_Change**
SalVentAttnA	173	27	146	SomMotA	210	36	174	ContB	149	43	106
ContB	167	26	141	Brainstem	16	8	8	SomMotA	236	136	100
SalVentAttnB	105	6	99	VisCent	7	4	3	SalVentAttnA	151	60	91
DefaultB	71	7	64	LimbicB	3	0	3	SalVentAttnB	90	16	74
Caudate nucleu	82	24	58	TempPar	3	2	1	DorsAttnA	50	1	49
DorsAttnA	50	1	49	LimbicA	1	0	1	TempPar	50	1	49
TempPar	49	0	49	DorsAttnA	2	2	0	LimbicB	42	0	42
LimbicB	42	0	42	Thalamus	2	5	-3	VisCent	32	1	31
VisCent	30	1	29	DefaultA	1	13	-12	Caudate nucleu	72	43	29
ContA	93	66	27	SalVentAttnB	25	42	-17	DefaultB	54	27	27
LimbicA	21	0	21	Caudate nucleu	22	47	-25	LimbicA	21	0	21
DefaultA	25	8	17	ContB	13	44	-31	VisPeri	17	0	17
VisPeri	17	0	17	SomMotB	18	56	-38	Brainstem	26	17	9
SomMotA	173	162	11	DefaultB	8	46	-38	Thalamus	14	8	6
Brainstem	23	16	7	DorsAttnB	11	60	-49	DefaultA	20	15	5
Thalamus	14	8	6	SalVentAttnA	27	83	-56	DefaultC	4	0	4
DefaultC	4	0	4	ContA	15	76	-61	ContC	3	0	3
ContC	3	0	3					ContA	65	84	-19
DorsAttnB	45	56	-11					SomMotB	57	110	-53
SomMotB	57	78	-21					DorsAttnB	35	94	-59
**Patient 3**											
**RSN**	**Increasing**	**Decreasing**	**Net_Change**	**RSN**	**Increasing**	**Decreasing**	**Net_Change**	**RSN**	**Increasing**	**Decreasing**	**Net_Change**
Caudate nucleu	85	0	85	Caudate nucleu	50	0	50	Caudate nucleu	100	0	100
Brainstem	51	0	51	Brainstem	45	1	44	Brainstem	64	0	64
SomMotA	35	0	35	SomMotA	31	0	31	SomMotA	41	0	41
DorsAttnA	21	0	21	DorsAttnA	17	0	17	DorsAttnA	27	0	27
SalVentAttnA	17	0	17	DorsAttnB	12	0	12	SalVentAttnA	18	0	18
DorsAttnB	10	0	10	SalVentAttnA	7	0	7	DorsAttnB	17	0	17
SomMotB	9	0	9	SomMotB	5	0	5	SomMotB	11	0	11
ContA	8	0	8	TempPar	5	0	5	ContA	8	0	8
ContB	6	0	6	ContA	4	0	4	ContB	7	0	7
SalVentAttnB	5	0	5	ContC	4	0	4	ContC	7	0	7
LimbicA	5	0	5	SalVentAttnB	2	0	2	TempPar	7	0	7
ContC	5	0	5	LimbicA	2	0	2	SalVentAttnB	5	0	5
TempPar	5	0	5	ContB	2	0	2	LimbicA	5	0	5
VisCent	4	0	4	VisCent	1	0	1	VisCent	4	0	4
DefaultA	4	0	4	VisPeri	1	0	1	DefaultA	3	0	3
DefaultB	2	0	2	DefaultC	1	0	1	DefaultB	2	0	2
VisPeri	1	0	1	DefaultA	1	1	0	VisPeri	1	0	1
DefaultC	1	0	1					DefaultC	1	0	1
**Patient 4**											
**RSN**	**Increasing**	**Decreasing**	**Net_Change**	**RSN**	**Increasing**	**Decreasing**	**Net_Change**	**RSN**	**Increasing**	**Decreasing**	**Net_Change**
Caudate nucleu	28	11	17	Caudate nucleu	37	1	36	Caudate nucleu	44	3	41
SomMotA	14	1	13	SomMotA	11	0	11	SomMotA	19	0	19
Brainstem	5	0	5	SomMotB	8	0	8	SomMotB	7	0	7
SalVentAttnA	5	1	4	DorsAttnB	8	0	8	DorsAttnB	7	1	6
ContA	5	2	3	SalVentAttnA	3	0	3	SalVentAttnA	6	0	6
DorsAttnB	4	1	3	ContA	2	0	2	ContA	6	1	5
SomMotB	3	2	1	DefaultB	2	0	2	Brainstem	5	0	5
ContB	1	0	1	TempPar	2	0	2	DefaultB	2	0	2
DefaultB	1	2	-1	VisCent	1	0	1	ContB	1	0	1
VisCent	0	1	-1	DefaultA	0	1	-1	TempPar	1	0	1
TempPar	0	1	-1					DefaultA	0	1	-1

Supplementary Table 3. Network-specific substantial connectivity strength changes in white matter connections, classified as >10% positive or negative change from baseline of each patient. For Patients 1-4, the difference between connections with substantial increases and decreases were calculated for each resting-state network across the post-stroke recovery stage transitions: S1-S2 (<1 week to ~1 month), S2-S3 (~1 to ~3 months), and S1-S3 (<1 week to ~3 months).

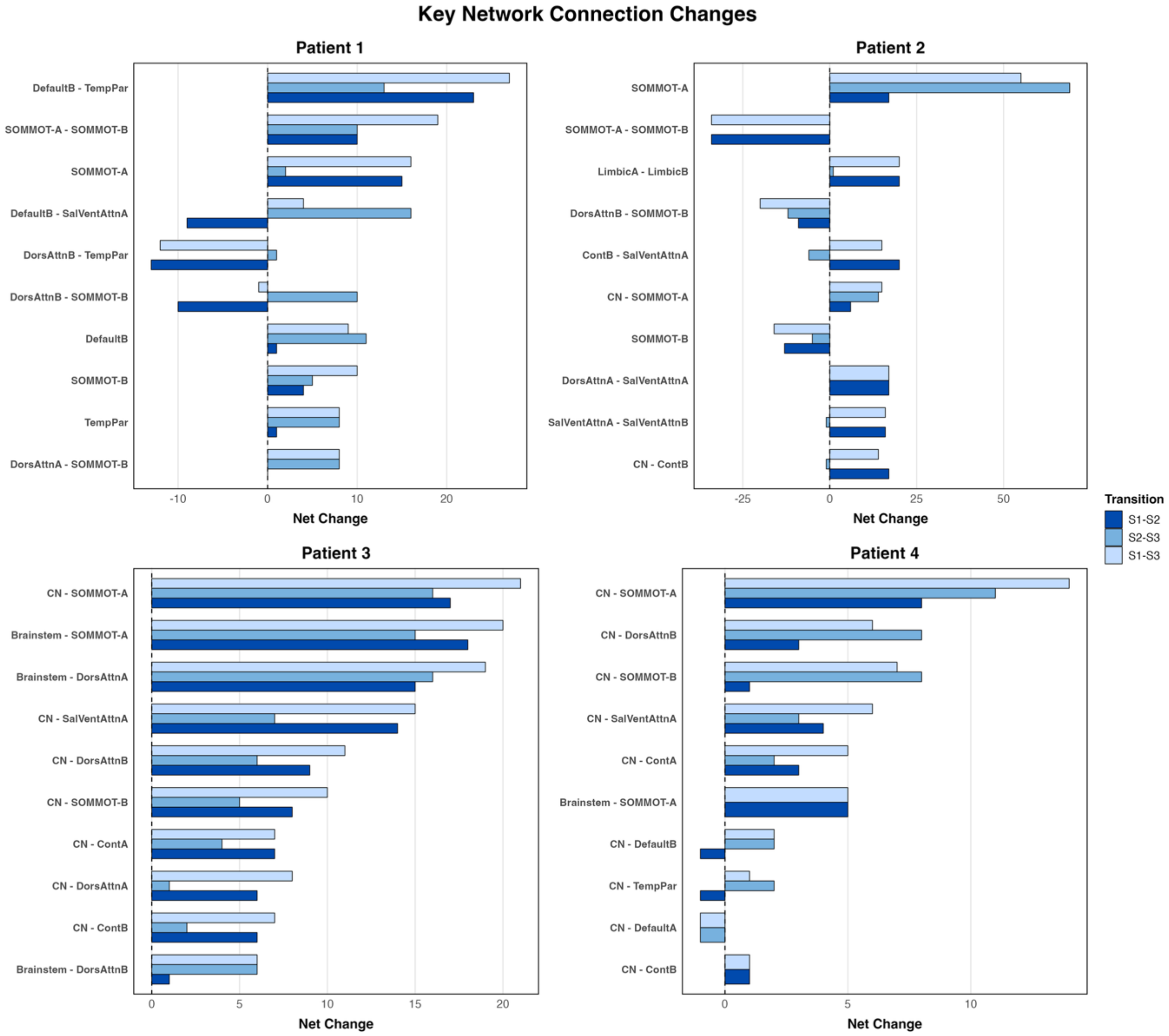

Supplementary Figure 3. Key network connection changes. For Patients 1-4, the top 10 unique network pairs with the highest absolute change in connectivity strength are displayed across post-stroke recovery stages: S1-S2 (<1 week to ~1 month), S2-S3 (~1 to ~3 months), and S1-S3 (<1 week to ~3 months). The total absolute change for each unique connection was calculated by summing the absolute values of net changes across all three recovery stages and all connections were than ranked by this cumulative metric to identify the connections based on their overall impact across the entire recovery period.

	Patient 1				
	**Threshold (%)**	**Total**	**Increases**	**Decreases**	**Strengthening (%)**
**S1-S2**	5	324	167	157	51.5
	10	311	156	155	50.2
	20	291	140	151	48.1
**S2-S3**	5	239	233	6	97.5
	10	233	227	6	97.4
	20	223	219	4	98.2
**S1-S3**	5	400	295	105	73.8
	10	387	284	103	73.4
	20	374	271	103	72.5
	**Patient 2**				
	**Threshold (%)**	**Total**	**Increases**	**Decreases**	**Strengthening (%)**
**S1-S2**	5	892	645	247	72.3
	10	865	622	243	71.9
	20	809	586	223	72.4
**S2-S3**	5	479	201	278	42
	10	454	192	262	42.3
	20	407	167	240	41
**S1-S3**	5	950	615	335	64.7
	10	922	594	328	64.4
	20	866	558	308	64.4
	**Patient 3**				
	**Threshold (%)**	**Total**	**Increases**	**Decreases**	**Strengthening (%)**
**S1-S2**	5	150	150	0	100
	10	137	137	0	100
	20	114	114	0	100
**S2-S3**	5	103	102	1	99
	10	96	95	1	99
	20	74	73	1	98.6
**S1-S3**	5	178	178	0	100
	10	164	164	0	100
	20	145	145	0	100
	**Patient 4**				
	**Threshold (%)**	**Total**	**Increases**	**Decreases**	**Strengthening (%)**
**S1-S2**	5	51	37	14	72.5
	10	44	33	11	75
	20	34	28	6	82.4
**S2-S3**	5	44	43	1	97.7
	10	38	37	1	97.4
	20	28	27	1	96.4
**S1-S3**	5	60	56	4	93.3
	10	52	49	3	94.2
	20	42	39	3	92.9

Supplementary Table 4. Sensitivity analysis of edge-level white matter connectivity changes across 5%, 10%, and 20% thresholds. For Patients 1-4, the total number of substantial connection changes include the following: Re-emerging connections (absent at the earlier timepoint but present at the later timepoint, gained connection), disconnections (present at the earlier timepoint but absent at the later timepoint, lost connection), and persistent connections exceeding the percent-change threshold (present at both timepoints with a relative percentage change from baseline). Connectivity increases include persistent increases and gained connections, connectivity decreases include persistent decreases and lost connections. Post-stroke recovery stages refer to: S1-S2 (<1 week to ~1 month), S2-S3 (~1 to ~3 months), and S1-S3 (<1 week to ~3 months).

	S1-S2				S2-S3					S1-S3				
Patient 1														
**Threshold (%)**	**RSN**	**Increasing**	**Decreasing**	**Net_Change**	**Threshold (%**	**RSN**	**Increasing**	**Decreasing**	**Net_Change**	**Threshold (%**	**RSN**	**Increasing**	**Decreasing**	**Net_Change**
**5**	SomMotA	59	8	51	**5**	SomMotA	24	0	24	**5**	SomMotA	74	3	71
**10**	SomMotA	52	8	44	**10**	SomMotA	23	0	23	**10**	SomMotA	67	3	64
**20**	SomMotA	43	8	35	**20**	SomMotA	22	0	22	**20**	SomMotA	59	3	56
**5**	SomMotB	54	44	10	**5**	SomMotB	75	0	75	**5**	SomMotB	100	24	76
**10**	SomMotB	51	43	8	**10**	SomMotB	75	0	75	**10**	SomMotB	97	23	74
**20**	SomMotB	47	42	5	**20**	SomMotB	72	0	72	**20**	SomMotB	95	23	72
**5**	SalVentAttnA	27	52	-25	**5**	SalVentAttnA	50	4	46	**5**	SalVentAttnA	54	40	14
**10**	SalVentAttnA	23	52	-29	**10**	SalVentAttnA	48	4	44	**10**	SalVentAttnA	50	40	10
**20**	SalVentAttnA	23	52	-29	**20**	SalVentAttnA	48	3	45	**20**	SalVentAttnA	50	40	10
**5**	SalVentAttnB	8	10	-2	**5**	SalVentAttnB	16	0	16	**5**	SalVentAttnB	16	6	10
**10**	SalVentAttnB	8	10	-2	**10**	SalVentAttnB	16	0	16	**10**	SalVentAttnB	16	6	10
**20**	SalVentAttnB	6	9	-3	**20**	SalVentAttnB	15	0	15	**20**	SalVentAttnB	16	6	10
**5**	DorsAttnA	11	17	-6	**5**	DorsAttnA	23	2	21	**5**	DorsAttnA	29	13	16
**10**	DorsAttnA	11	17	-6	**10**	DorsAttnA	23	2	21	**10**	DorsAttnA	29	13	16
**20**	DorsAttnA	11	16	-5	**20**	DorsAttnA	23	1	22	**20**	DorsAttnA	29	13	16
**5**	DorsAttnB	21	60	-39	**5**	DorsAttnB	35	0	35	**5**	DorsAttnB	41	52	-11
**10**	DorsAttnB	20	60	-40	**10**	DorsAttnB	34	0	34	**10**	DorsAttnB	40	52	-12
**20**	DorsAttnB	20	60	-40	**20**	DorsAttnB	34	0	34	**20**	DorsAttnB	40	52	-12
Patient 2														
**Threshold (%)**	**RSN**	**Increasing**	**Decreasing**	**Net_Change**	**Threshold (%**	**RSN**	**Increasing**	**Decreasing**	**Net_Change**	**Threshold (%**	**RSN**	**Increasing**	**Decreasing**	**Net_Change**
**5**	SalVentAttnA	173	29	144	**5**	SalVentAttnA	28	89	-61	**5**	SalVentAttnA	151	64	87
**10**	SalVentAttnA	173	27	146	**10**	SalVentAttnA	27	83	-56	**10**	SalVentAttnA	151	60	91
**20**	SalVentAttnA	168	22	146	**20**	SalVentAttnA	20	77	-57	**20**	SalVentAttnA	150	57	93
**5**	SalVentAttnB	109	6	103	**5**	SalVentAttnB	25	42	-17	**5**	SalVentAttnB	94	17	77
**10**	SalVentAttnB	105	6	99	**10**	SalVentAttnB	25	42	-17	**10**	SalVentAttnB	90	16	74
**20**	SalVentAttnB	99	5	94	**20**	SalVentAttnB	21	42	-21	**20**	SalVentAttnB	85	15	70
**5**	SomMotA	174	163	11	**5**	SomMotA	216	36	180	**5**	SomMotA	238	136	102
**10**	SomMotA	173	162	11	**10**	SomMotA	210	36	174	**10**	SomMotA	236	136	100
**20**	SomMotA	167	159	8	**20**	SomMotA	197	34	163	**20**	SomMotA	223	133	90
**5**	SomMotB	57	78	-21	**5**	SomMotB	19	61	-42	**5**	SomMotB	57	113	-56
**10**	SomMotB	57	78	-21	**10**	SomMotB	18	56	-38	**10**	SomMotB	57	110	-53
**20**	SomMotB	55	76	-21	**20**	SomMotB	16	48	-32	**20**	SomMotB	55	102	-47
**5**	DorsAttnA	51	1	50	**5**	DorsAttnA	2	2	0	**5**	DorsAttnA	51	1	50
**10**	DorsAttnA	50	1	49	**10**	DorsAttnA	2	2	0	**10**	DorsAttnA	50	1	49
**20**	DorsAttnA	42	1	41	**20**	DorsAttnA	2	2	0	**20**	DorsAttnA	42	1	41
**5**	DorsAttnB	49	56	-7	**5**	DorsAttnB	14	64	-50	**5**	DorsAttnB	39	96	-57
**10**	DorsAttnB	45	56	-11	**10**	DorsAttnB	11	60	-49	**10**	DorsAttnB	35	94	-59
**20**	DorsAttnB	40	52	-12	**20**	DorsAttnB	11	56	-45	**20**	DorsAttnB	31	90	-59
Patient 3														
**Threshold (%)**	**RSN**	**Increasing**	**Decreasing**	**Net_Change**	**Threshold (%**	**RSN**	**Increasing**	**Decreasing**	**Net_Change**	**Threshold (%**	**RSN**	**Increasing**	**Decreasing**	**Net_Change**
**5**	Caudate nucleu	97	0	97	**5**	Caudate nucleu	56	0	56	**5**	Caudate nucleu	112	0	112
**10**	Caudate nucleu	85	0	85	**10**	Caudate nucleu	50	0	50	**10**	Caudate nucleu	100	0	100
**20**	Caudate nucleu	63	0	63	**20**	Caudate nucleu	34	0	34	**20**	Caudate nucleu	82	0	82
**5**	SomMotA	36	0	36	**5**	SomMotA	33	0	33	**5**	SomMotA	43	0	43
**10**	SomMotA	35	0	35	**10**	SomMotA	31	0	31	**10**	SomMotA	41	0	41
**20**	SomMotA	33	0	33	**20**	SomMotA	26	0	26	**20**	SomMotA	40	0	40
**5**	SomMotB	9	0	9	**5**	SomMotB	5	0	5	**5**	SomMotB	11	0	11
**10**	SomMotB	9	0	9	**10**	SomMotB	5	0	5	**10**	SomMotB	11	0	11
**20**	SomMotB	9	0	9	**20**	SomMotB	4	0	4	**20**	SomMotB	11	0	11
**5**	DorsAttnA	24	0	24	**5**	DorsAttnA	18	0	18	**5**	DorsAttnA	30	0	30
**10**	DorsAttnA	21	0	21	**10**	DorsAttnA	17	0	17	**10**	DorsAttnA	27	0	27
**20**	DorsAttnA	18	0	18	**20**	DorsAttnA	15	0	15	**20**	DorsAttnA	23	0	23
**5**	DorsAttnB	10	0	10	**5**	DorsAttnB	14	0	14	**5**	DorsAttnB	17	0	17
**10**	DorsAttnB	10	0	10	**10**	DorsAttnB	12	0	12	**10**	DorsAttnB	17	0	17
**20**	DorsAttnB	8	0	8	**20**	DorsAttnB	11	0	11	**20**	DorsAttnB	14	0	14
**5**	SalVentAttnA	18	0	18	**5**	SalVentAttnA	7	0	7	**5**	SalVentAttnA	19	0	19
**10**	SalVentAttnA	17	0	17	**10**	SalVentAttnA	7	0	7	**10**	SalVentAttnA	18	0	18
**20**	SalVentAttnA	12	0	12	**20**	SalVentAttnA	3	0	3	**20**	SalVentAttnA	15	0	15
**5**	SalVentAttnB	5	0	5	**5**	SalVentAttnB	3	0	3	**5**	SalVentAttnB	5	0	5
**10**	SalVentAttnB	5	0	5	**10**	SalVentAttnB	2	0	2	**10**	SalVentAttnB	5	0	5
**20**	SalVentAttnB	2	0	2	**20**	SalVentAttnB	1	0	1	**20**	SalVentAttnB	4	0	4
Patient 4														
**Threshold (%)**	**RSN**	**Increasing**	**Decreasing**	**Net_Change**	**Threshold (%**	**RSN**	**Increasing**	**Decreasing**	**Net_Change**	**Threshold (%**	**RSN**	**Increasing**	**Decreasing**	**Net_Change**
**5**	Caudate nucleu	31	14	17	**5**	Caudate nucleu	43	1	42	**5**	Caudate nucleu	50	4	46
**10**	Caudate nucleu	28	11	17	**10**	Caudate nucleu	37	1	36	**10**	Caudate nucleu	44	3	41
**20**	Caudate nucleu	24	6	18	**20**	Caudate nucleu	27	1	26	**20**	Caudate nucleu	35	3	32
**5**	SomMotA	15	2	13	**5**	SomMotA	14	0	14	**5**	SomMotA	22	0	22
**10**	SomMotA	14	1	13	**10**	SomMotA	11	0	11	**10**	SomMotA	19	0	19
**20**	SomMotA	13	1	12	**20**	SomMotA	10	0	10	**20**	SomMotA	17	0	17
**5**	SomMotB	3	2	1	**5**	SomMotB	8	0	8	**5**	SomMotB	7	0	7
**10**	SomMotB	3	2	1	**10**	SomMotB	8	0	8	**10**	SomMotB	7	0	7
**20**	SomMotB	3	2	1	**20**	SomMotB	7	0	7	**20**	SomMotB	6	0	6
**5**	SalVentAttnA	5	2	3	**5**	SalVentAttnA	4	0	4	**5**	SalVentAttnA	6	0	6
**10**	SalVentAttnA	5	1	4	**10**	SalVentAttnA	3	0	3	**10**	SalVentAttnA	6	0	6
**20**	SalVentAttnA	2	0	2	**20**	SalVentAttnA	1	0	1	**20**	SalVentAttnA	4	0	4
**5**	SalVentAttnB	2	0	2	**5**	SalVentAttnB	0	0	0	**5**	SalVentAttnB	2	0	2
**10**	SalVentAttnB	0	0	0	**10**	SalVentAttnB	0	0	0	**10**	SalVentAttnB	0	0	0
**20**	SalVentAttnB	0	0	0	**20**	SalVentAttnB	0	0	0	**20**	SalVentAttnB	0	0	0
**5**	DorsAttnB	4	2	2	**5**	DorsAttnB	8	0	8	**5**	DorsAttnB	8	1	7
**10**	DorsAttnB	4	1	3	**10**	DorsAttnB	8	0	8	**10**	DorsAttnB	7	1	6
**20**	DorsAttnB	4	1	3	**20**	DorsAttnB	4	0	4	**20**	DorsAttnB	5	1	4

Supplementary Table 5. Network-specific substantial connectivity strength changes in white matter connections, tested as >5%, >10%, and >20% positive or negative change from baseline of each patient. For Patients 1-4, the difference between connections with substantial increases and decreases were calculated for the somatomotor, attention, and caudate nucleus resting-state networks across the post-stroke recovery stage transitions: S1-S2 (<1 week to ~1 month), S2-S3 (~1 to ~3 months), and S1-S3 (<1 week to ~3 months). Included networks are: SomMotA: Somatomotor-A, SomMotB; Somatomotor-B, SalVentAttnA: Salience/Ventral Attention-A, SalVentAttnB: Salience/Ventral Attention-B, DorsAttnA: Dorsal attention-A, DorsAttnB: Dorsal attention-B, caudate nucleus.

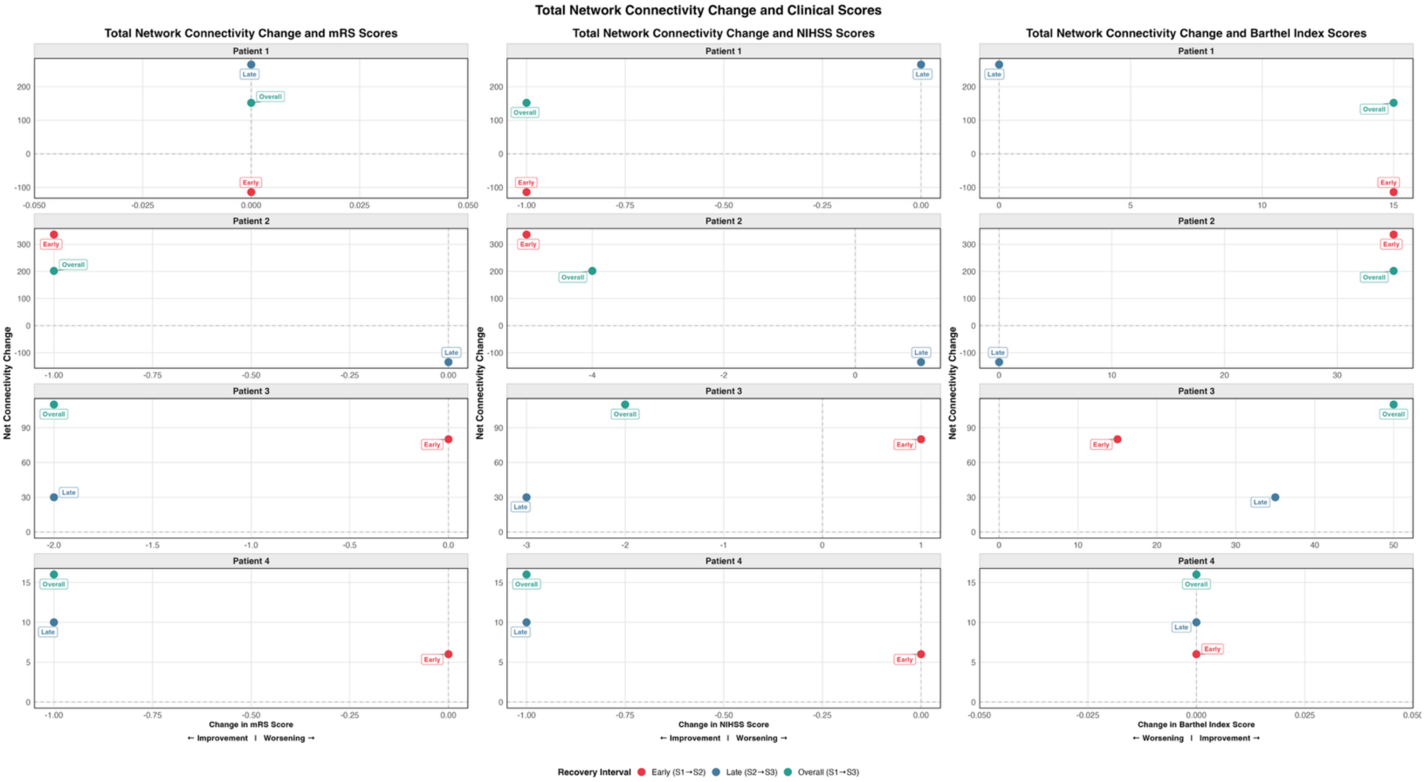

Supplementary Figure 4. Total network white matter connectivity changes and clinical outcome scores. For Patients 1-4, net connectivity changes are computed as the number of re-emerging connections (absent at the earlier timepoint but present at the later timepoint) minus disconnections (present at the earlier timepoint but absent at the later timepoint) across the post-stroke recovery stage transitions: Early S1-S2 (<1 week to ~1 month, in red), Late S2-S3 (~1 to ~3 months, in blue), and Overall S1-S3 (<1 week to ~3 months, in green). First column includes modified Rankin Scale (mRS) scoles, higher scores indicate worse global disability level. Second column includes National Institutes of Health Stroke Scale (NIHSS) scores, higher scores indicate worse clinical stroke status. Third column includes Barthel Index scores, higher scores indicate better independence in activities of daily living.

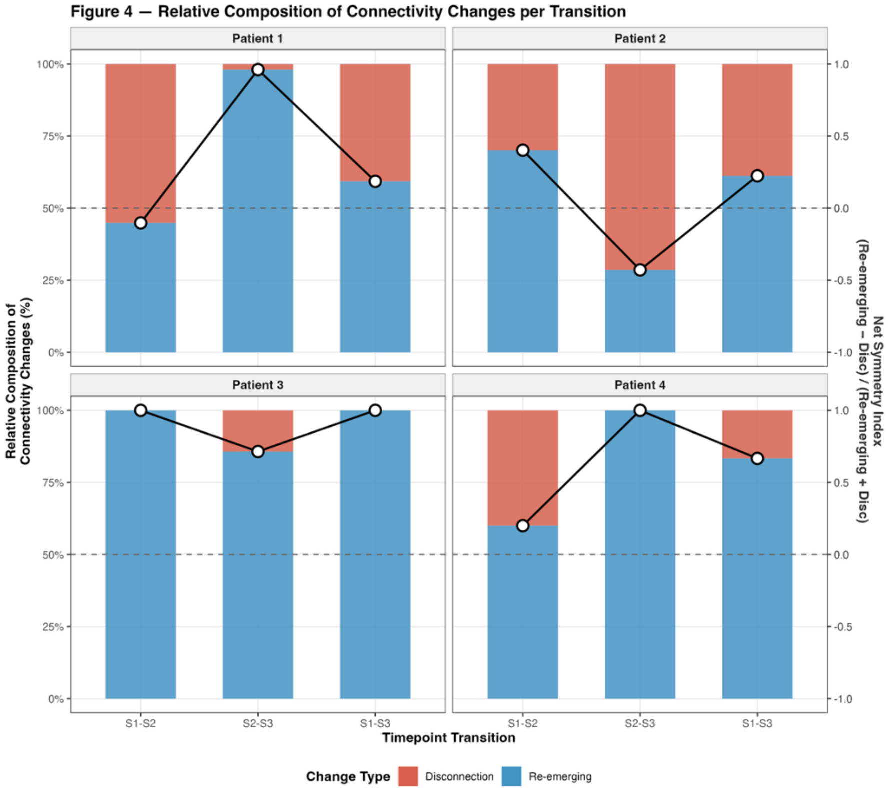

Supplementary Figure 5. Relative composition of disconnections (red) and re-emerging connections (blue) as a parcentage total binary connectivity changes (re-emerging connections + disconnections). For Patients 1 – 4, the black line shows net symmetry calculated as (re-emerging – disconnections) / (re-emerging + disconnections), where positive values indicate re-emerging-dominant transitions and negative values indicate disconnection-dominant transitions across the post-stroke recovery stage transitions: S1-S2 (<1 week to ~1 month), S2-S3 (~1 to ~3 months), and S1-S3 (<1 week to ~3 months).

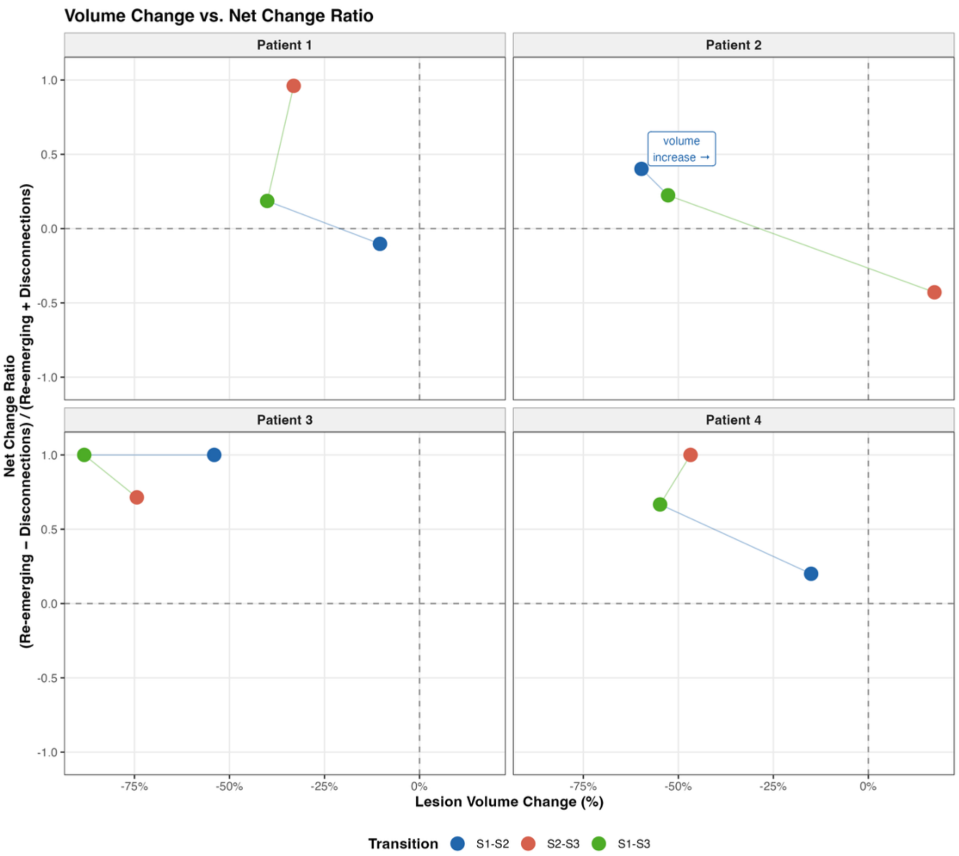

Supplementary Figure 6. Lesion volume change from previous timepoint and net connectivity change ratio. For Patients 1 – 4, net connectivity change ratio was calculated as (re-emerging connections – disconnections) / (re-emerging connections + disconnections), where re-emerging connections are connections absent at the earlier timepoint but present at the later timepoint and disconnections are connections present at the earlier timepoint but absent at the later timepoint, across the post-stroke recovery stage transitions: S1-S2 (<1 week to ~1 month, in blue), S2-S3 (~1 to ~3 months, in red), and S1-S3 (<1 week to ~3 months, in green). For the lesion volume change, values lower than 0 indicate volume reduction and greater than 0 indicate volume expansion. For the net change ratio, values above 0 indicate re-emergence dominance and below 0 indicates disconnection dominance.

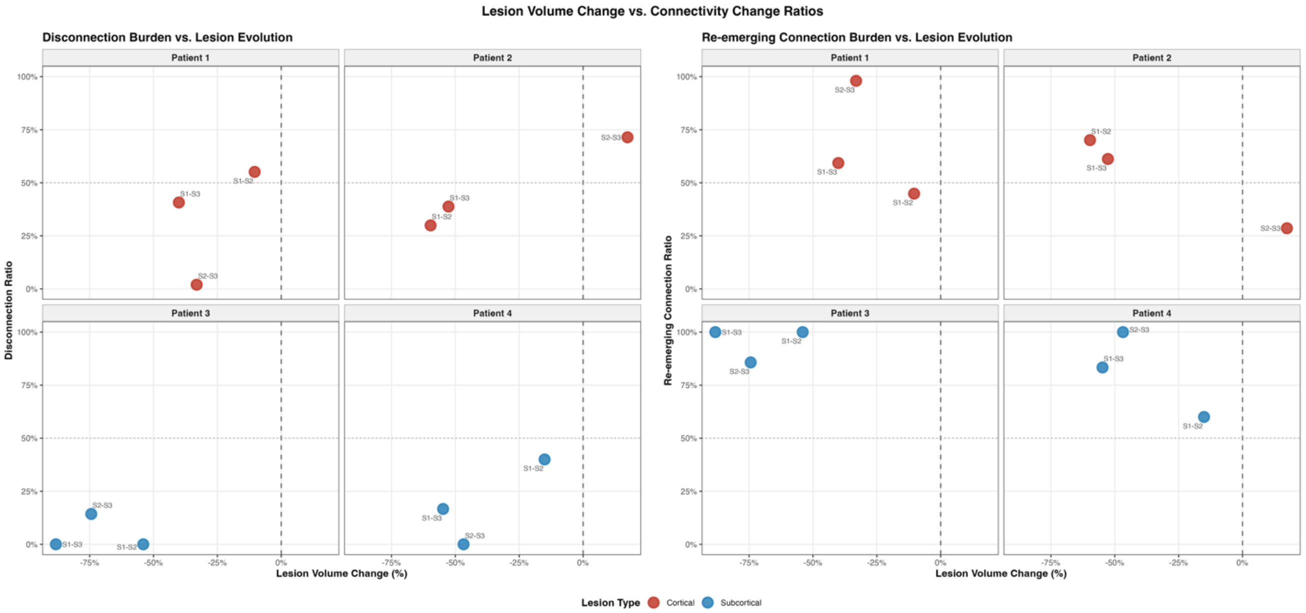

Supplementary Figure 7. Lesion volume change from previous timepoint and connectivity change ratios. For Patients 1 – 4, disconnection ratio is calculated as the number of disconnections / (disconnections + re-emerging connections) and re-emerging connection ratio is re-emerging connections / (disconnections + re-emerging connections) across the post-stroke recovery stage transitions: S1-S2 (<1 week to ~1 month), S2-S3 (~1 to ~3 months), and S1-S3 (<1 week to ~3 months). For the lesion volume change, values lower than 0 indicate volume reduction and greater than 0 indicate volume expansion.

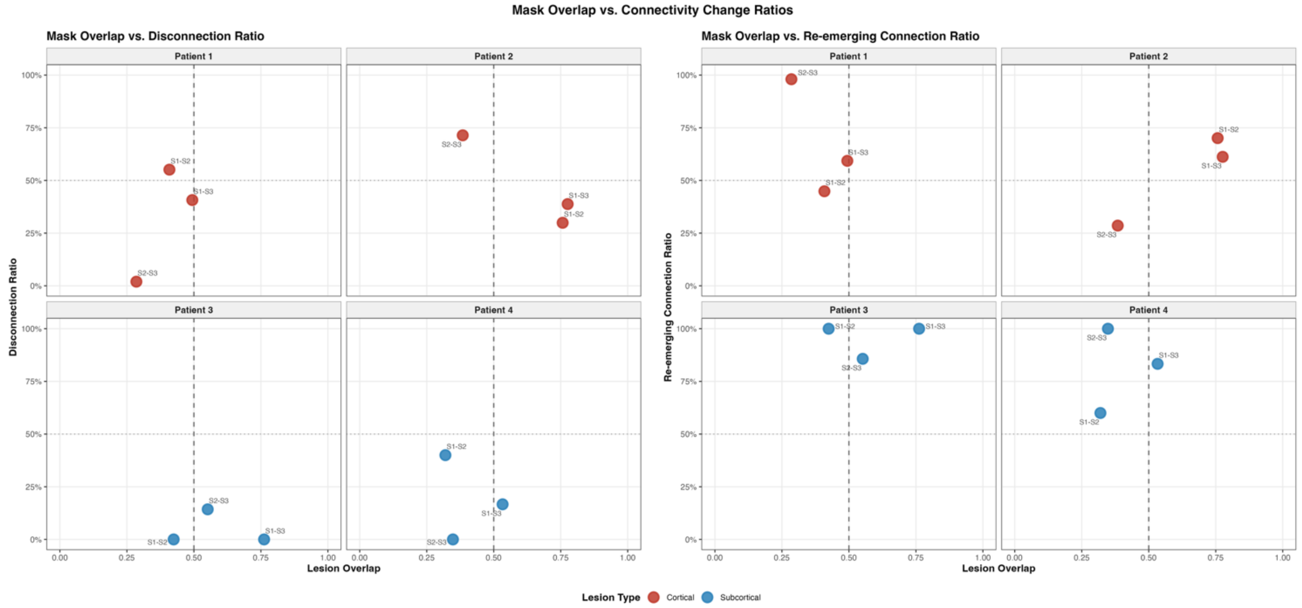

Supplementary Figure 8. Lesion mask overlap and conectivity change ratios. For Patients 1 – 4, lesion overlap is calculated as (1-DSC), disconnection ratio is calculated as the number of disconnections / (disconnections + re-emerging connections) and re-emerging connection ratio is re-emerging connections / (disconnections + re-emerging connections) across the post-stroke recovery stage transitions: S1-S2 (<1 week to ~1 month), S2-S3 (~1 to ~3 months), and S1-S3 (<1 week to ~3 months). For the lesion overlap, higher values indicate greater spatial difference between timepoints and lower values indicate greater spatial similarity. *DSC: Dice similarity coefficient.*

References

1. Iandolo R, Avci E, Bommarito G, Sandvig I, Rohweder G, Sandvig A. Characterizing upper extremity fine motor function in the presence of white matter hyperintensities: A 7 T MRI cross-sectional study in older adults. NeuroImage: Clinical. 2024;41:103569.

2. Gulban OF, Nielson D, Poldrack R, Lee john, Gorgolewski C, Vanessasaurus, et al. poldracklab/pydeface: v2.0.0. 2019 Oct; Available from: https://zenodo.org/record/3524401

3. Kashyap S, Ivanov D, Havlicek M, Huber L, Poser BA, Uludağ K. Sub-millimetre resolution laminar fMRI using Arterial Spin Labelling in humans at 7 T. Plos one. 2021;16(4):e0250504.

4. Choi US, Kawaguchi H, Matsuoka Y, Kober T, Kida I. Brain tissue segmentation based on MP2RAGE multi-contrast images in 7 T MRI. PloS one. 2019;14(2):e0210803.

5. Greve DN, Fischl B. Accurate and robust brain image alignment using boundary-based registration. NeuroImage. 2009;48(1):63–72.

6. Isensee F, Schell M, Pflueger I, Brugnara G, Bonekamp D, Neuberger U, et al. Automated brain extraction of multisequence MRI using artificial neural networks. Human brain mapping. 2019;40(17):4952–64.

7. de Hollander G, van der Zwaag W, Qian C, Zhang P, Knapen T. Ultra-high field fMRI reveals origins of feedforward and feedback activity within laminae of human ocular dominance columns. NeuroImage. 2021;228:117683.

8. Tournier JD, Smith R, Raffelt D, Tabbara R, Dhollander T, Pietsch M, et al. MRtrix3: A fast, flexible and open software framework for medical image processing and visualisation. NeuroImage. 2019 Nov;202:116137.

9. Jenkinson M, Bannister P, Brady M, Smith S. Improved Optimization for the Robust and Accurate Linear Registration and Motion Correction of Brain Images. NeuroImage. 2002 Oct;17(2):825–41.

10. Tustison NJ, Cook PA, Holbrook AJ, Johnson HJ, Muschelli J, Devenyi GA, et al. The ANTsX ecosystem for quantitative biological and medical imaging. Sci Rep. 2021 Apr 27;11(1):9068.

11. Avants BB, Epstein CL, Grossman M, Gee JC. Symmetric diffeomorphic image registration with cross-correlation: evaluating automated labeling of elderly and neurodegenerative brain. Medical image analysis. 2008;12(1):26–41.

12. Klein A, Andersson J, Ardekani BA, Ashburner J, Avants B, Chiang MC, et al. Evaluation of 14 nonlinear deformation algorithms applied to human brain MRI registration. NeuroImage. 2009 July;46(3):786–802.

13. Yeh FC. DSI Studio: an integrated tractography platform and fiber data hub for accelerating brain research. Nat Methods. 2025 Aug;22(8):1617–9.
